# Human pancreatic beta-like cells converted from fibroblasts

**DOI:** 10.1038/ncomms10080

**Published:** 2016-01-06

**Authors:** Saiyong Zhu, Holger A. Russ, Xiaojing Wang, Mingliang Zhang, Tianhua Ma, Tao Xu, Shibing Tang, Matthias Hebrok, Sheng Ding

**Affiliations:** 1 Gladstone Institute of Cardiovascular Disease, University of California, San Francisco, California 94158, USA; 2 Diabetes Center, Department of Medicine, University of California, San Francisco, San Francisco, California 94143, USA; 3 Department of Pharmaceutical Chemistry, University of California San Francisco, 600 16th Street, San Francisco, California 94158, USA

## Abstract

Pancreatic beta cells are of great interest for biomedical research and regenerative medicine. Here we show the conversion of human fibroblasts towards an endodermal cell fate by employing non-integrative episomal reprogramming factors in combination with specific growth factors and chemical compounds. On initial culture, converted definitive endodermal progenitor cells (cDE cells) are specified into posterior foregut-like progenitor cells (cPF cells). The cPF cells and their derivatives, pancreatic endodermal progenitor cells (cPE cells), can be greatly expanded. A screening approach identified chemical compounds that promote the differentiation and maturation of cPE cells into functional pancreatic beta-like cells (cPB cells) *in vitro*. Transplanted cPB cells exhibit glucose-stimulated insulin secretion *in vivo* and protect mice from chemically induced diabetes. In summary, our study has important implications for future strategies aimed at generating high numbers of functional beta cells, which may help restoring normoglycemia in patients suffering from diabetes.

## Abstract (web summary)

Insulin-producing pancreatic beta cells, generated *in vitro*, could lead to new anti-diabetic therapies. Here, Zhu *et al*. convert human fibroblasts into endodermal progenitors that differentiate *in vitro* into glucose-responsive beta-like cells that, following transplantation in mice, protect from diabetes.

## Graphical abstract

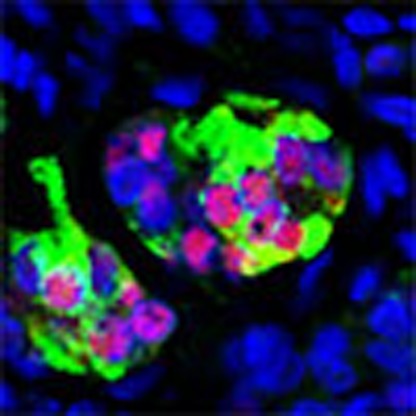

Generation of functional pancreatic insulin-producing beta cells for the effective treatment of diabetes is a key area of translational research. Stepwise differentiation protocols have been devised to guide the differentiation of human embryonic stem cells (hESCs)^1^, and more recently induced pluripotent stem cells (iPSCs)^2^, into definitive endoderm, primitive gut tube endoderm, posterior foregut endoderm and pancreatic endoderm (PE). These hESC-derived PE cells can mature into functional beta-like cells *in vivo* after prolonged periods following transplantation into immunodeficient mice^3^,^4^,^5^. More recently, improved differentiation protocols have been described that allow the formation of functional beta-like cells from hESCs under cell culture conditions^6^,^7^,^8^. While these findings are encouraging, several challenges remain and significant efforts have been directed towards further improvement of differentiation conditions^9^,^10^,^11^,^12^,^13^, the expansion of cells at different progenitor stages^14^,^15^ and the purification of target cell populations^16^ to obtain sufficient quantities of functional pancreatic beta cells.

An alternative strategy previously employed is the conversion of fibroblasts towards lineage-specific proliferative progenitors *in vitro*^17^,^18^,^19^. The benefit of such an approach is the ability to generate large numbers of cells suitable for any downstream assays and applications before final differentiation into the desired cell type *in vitro* or *in vivo*. We previously devised a Cell-Activation and Signalling-Directed (CASD) lineage conversion strategy, which employs transient overexpression of iPSC-transcription factors in conjunction with lineage-specific soluble signals to reprogramme somatic cells into diverse lineage-specific cell types without first establishing a pluripotent state^20^,^21^. Using this method, we and other groups have demonstrated the successful reprogramming of fibroblasts and other cells into cardiac cells^22^,^23^, neural progenitors^24^,^25^,^26^, angioblast-like progenitors^27^, endothelial cells^28^ and hepatocytes^29^. In addition, we recently demonstrated the generation of functional pancreatic beta-like cells from mouse fibroblasts using the CASD strategy^30^. However, successful translation of these approaches to generate functional human beta-like cells from fibroblasts that are capable of reversing diabetes in mouse models has not been demonstrated so far.

Here we demonstrate that human endodermal progenitor cells can be established from neonatal and adult human fibroblasts *in vitro* by employing a non-integrating reprogramming approach following the CASD transdifferentiation paradigm. We have identified conditions that allow great expansion of converted cells at distinct progenitor stages, including posterior foregut and PE. Converted pancreatic endodermal progenitors can be induced to efficiently differentiate into glucose-responsive beta-like cells *in vitro*. Beta-like cells transplanted into surrogate mice are functional *in vivo* and possess the ability to protect mice from diabetes. Therefore, these findings suggest an approach for the potential production of patient-specific insulin-producing cells to study relevant and unresolved questions in beta-cell biology.

## Results

### Converting fibroblasts into endodermal progenitor cells

**Figure 1:**
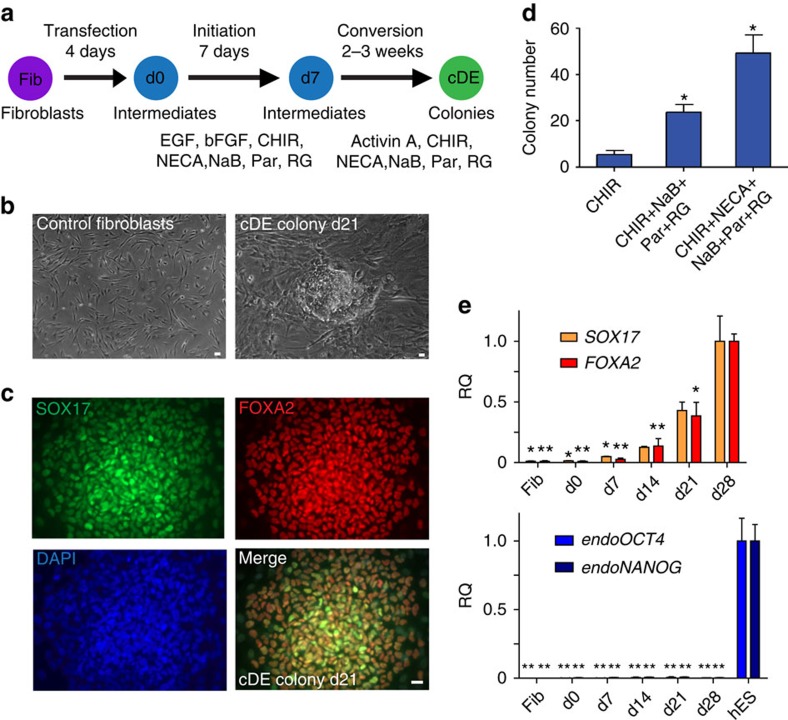
Conversion of human fibroblasts into definitive endodermal progenitor cells. (**a**) Schematic illustrating our strategy to convert human fibroblasts (Fib) into definitive endodermal progenitor cells (cDE cells) by combining non-integrating episomal reprogramming plasmids with specific initiation and conversion conditions. Epidermal growth factor (EGF), basic fibroblast growth factor (bFGF), CHIR 99021 (CHIR), 5′-N-ethylcarboxamidoadenosine (NECA), sodium butyrate (NaB), Parnate (Par) and RG108 (RG). (**b**) Bright-field images of control fibroblasts and a cDE colony at day 21. Scale bar, 20 μm. (**c**) Immunofluorescence staining of a representative cDE colony at day 21 for the endodermal progenitor markers SOX17 and FOXA2. Scale bar, 20 μm. (**d**) Small molecules sodium butyrate (NaB), Parnate (Par), RG108 (RG), CHIR99021 (CHIR) and 5′-N-ethylcarboxamidoadenosine (NECA) added to the basal condition further enhance endodermal reprogramming efficiency. Data represent the number of FOXA2-positive colonies scored at day 28 (mean values±s.e.m. of three experiments). Statistical significance calculated using two-tailed Student's *t*-test, compared with CHIR treatment. *P<0.05. (**e**) QPCR analyses of endodermal genes *SOX17* and *FOXA2*, and endogenous pluripotent genes *OCT4* (endo*OCT4*) and *NANOG* (endo*NANOG*) during the conversion process. Note that hESCs served as control. Mean value±s.e.m. are normalized to *Glyceraldehyde 3-phosphate dehydrogenase* (*GAPDH*) and relative to d28 cDE cultures and hESCs, respectively (*n*=3 experiments). Statistical significance calculated using two-tailed Student's *t*-test, compared with d28 cDE cultures and hESCs, respectively. *P<0.05, **P<0.01.

Human foreskin fibroblasts were transduced with non-integrating episomal reprogramming factors, OCT4, SOX2, KLF4 and a short hairpin RNA against p53^31^, recovered in fibroblast medium for 4 days, and cultured in initiation medium containing epidermal growth factor (EGF), basic fibroblast growth factor (bFGF) and CHIR99021 (an activator of WNT signalling) to support cell proliferation (Fig. 1a). After 7 days, the culture conditions were switched to endodermal conversion media containing high level of Activin A (100 ng ml^−1^) and CHIR99021 to establish converted definitive endodermal progenitor (cDE) cells, based on previous studies demonstrating key roles for the Activin A and WNT signalling pathways in endodermal fate decision *in vitro* and *in vivo*^32^,^33^,^34^. This basic endodermal conversion protocol gave rise to cell colonies with an epithelial morphology between days 21 to 28 (Fig. 1b). Colonies specifically stained for definitive endodermal progenitor markers SOX17 and FOXA2, but not for the pluripotency marker NANOG or primitive gut markers HNF6 and HNF4α (Fig. 1c; Supplementary Fig. 1a). As expected, control fibroblasts were negative for endodermal and pluripotency markers (Supplementary Fig. 1a). To increase the efficiency of our endodermal conversion protocol, we performed a small-scale screening of chemical compounds covering bioactive molecules capable of controlling cell fate^35^. We observed that combined addition of the epigenetic modulators: sodium butyrate (NaB, a histone deacetylase inhibitor), Parnate (Par, a histone demethylase inhibitor) and RG108 (RG, a DNA methylase inhibitor) could significantly improve the conversion efficiency by 2.5-fold (Fig. 1a,d). Additional screening using this improved condition revealed that 5′-N-ethylcarboxamidoadenosine (NECA, an adenosine agonist) could further increase the conversion rate by 2-fold, resulting in a 5-fold increase over the basal protocol (Fig. 1d). Notably, by employing this optimized condition, we could generate ∼50 FOXA2 and SOX17 double-positive cDE colonies from 400,000 human fibroblasts (Fig. 1d). Quantitative PCR (qPCR) analysis revealed that mRNA expression of fibroblast-specific markers, *THY1* and *COL1A1*, was rapidly downregulated during the first 7 days of reprogramming, suggesting a rapid dedifferentiation process (Supplementary Fig. 1b). Endodermal gene transcripts, *SOX17* and *FOXA2*, were gradually upregulated (Fig. 1e). In contrast, the expression of endogenous pluripotency gene transcripts *NANOG* and *OCT4* remained at very low levels during the conversion process (Fig. 1e; Supplementary Fig. 1c). Taken together, these data demonstrate that human fibroblasts can be converted into cDE cells using an episomal reprogramming system by employing the CASD transdifferentiation approach.

### Characterization of posterior foregut-like progenitor cells

**Figure 2:**
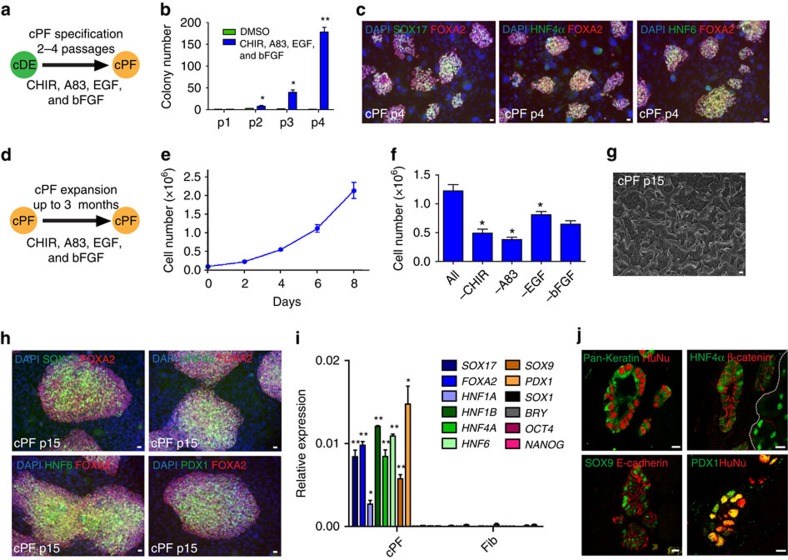
Specification, expansion and characterization of posterior foregut-like progenitor cells. (**a**) Schematic representation of culture conditions for the amplification of cDE cells that resulted in further specification into expandable posterior foregut-like progenitor cells (cPF cells). (**b**) Improved culture conditions allow amplification of cDE/cPF cell colonies. Mean values±s.e.m. represent three experiments. Statistical significance calculated using two-tailed Student's *t*-test, compared with DMSO controls. *P<0.05, **P<0.01. (**c**) Immunofluorescence analysis of colonies after four passages in improved expansion media suggested specification towards cPF cells. Scale bar, 20 μm. (**d**) Illustration of cPF expansion strategy. (**e**) Growth curve of cPF cells. Data from three experiments are shown as average±s.e.m. (**f**) All four media supplements—EGF, bFGF, A83-01 and CHIR99021—are important for cPF cell self-renewal (*n*=3 experiments). Statistical significance calculated using two-tailed Student's *t*-test, compared with ALL supplementation. *P<0.05. (**g**) Bright-field image of established cPF cells showing epithelial colony morphology. Scale bar, 20 μm. (**h**) Immunofluorescence staining of SOX17, FOXA2, HNF4α, HNF6 and PDX1 in cPF cells at passage 15. Scale bar, 20 μm. (**i**) QPCR analysis demonstrates the enrichment of transcripts for *SOX17*, *FOXA2*, *HNF1A*, *HNF1B*, *HNF4A*, *HNF6*, *SOX9* and *PDX1*, but not *SOX1, BRY, OCT4* and *NANOG* in p15 cPF cells. Mean values±s.e.m. are normalized to *GAPDH* relative to control fibroblasts. (*n*=3 experiments). Statistical significance calculated using two-tailed Student's *t*-test, compared with fibroblast controls. *P<0.05, **P<0.01. (**j**) Immunofluorescence analysis of cPF cell grafts shows epithelial structures that express E-cadherin, HNF4α, PDX1, SOX9 and pan-cytokeratin. Human nuclear antigen (HuNu) demonstrates the human cell origin. Scale bar, 20 μm.

Next, we attempted to serially expand the number of cDE colonies via treatment with media containing two small molecules, the WNT signalling activator CHIR99021 and the TGFβ signalling inhibitor A83-01 in conjunction with two growth factors EGF and bFGF that significantly promoted their expansion (Fig. 2a,b). Immunofluorescence analysis revealed strong expression of endodermal progenitor markers SOX17 and FOXA2, but also induction of primitive gut tube marker HNF4α and posterior foregut marker HNF6 (Fig. 2c), suggesting further specification towards posterior foregut-like progenitor cells (cPF cells). Employing these culture conditions, we established five cPF cell lines from four independent experiments employing human neonatal fibroblasts (Fig. 2d). cPF cells proliferated rapidly with an average doubling time of 2 days for up to 15 passages (Fig. 2e,g). All four media supplements were important for cPF cell self-renewal (Fig. 2f) and expanded cPF cells maintained their epithelial colony morphology, as well as posterior foregut-like phenotype as determined by immunofluorescence staining for SOX17, FOXA2, HNF4α, HNF6 and SOX9 (Fig. 2g,h; Supplementary Figs 2–4). During mouse embryonic development, Pdx1 expression is first detected at embryonic day 8.5 and marks the endodermal region that will give rise to the whole pancreas, as well as the common bile duct, distal stomach and duodenal epithelium^36^,^37^. Interestingly, we detect PDX1 protein (Fig. 2h). Consistently, qPCR analysis demonstrated the induced high-level expression of multiple posterior foregut progenitor gene transcripts, including *SOX17*, *FOXA2*, *HNF1A*, *HNF1B*, *HNF4A*, *HNF6*, *SOX9* and *PDX1* in cPF cells when compared with parental fibroblasts (Fig. 2i). In contrast, ectodermal marker gene *SOX1*, mesodermal marker gene *BRACHYURY*, and pluripotency marker genes *OCT4* and *NANOG* were not induced (Fig. 2i). Collectively, these data confirm the specific posterior foregut identity of cPF cells, which is further supported by our observations that cPF cells at both early and late passages possessed comparable capacities to differentiate towards the hepatic lineage (Supplementary Fig. 5b). Of note, cPF cells can be frozen and thawed with a recovery rate of 82.7±5.1% (*n*=3), an important advantage enabling many downstream assays. We further checked the genome stability of cPF cells using comparative genomic hybridization arrays and did not observe any gross chromosomal aberrations, but detected four copy number variants in one human neonatal fibroblast-derived cPF cell line (Supplementary Fig. 6). This result is consistent with a recent report demonstrating that long-term cultures of bipotent stem cells from adult human liver are genome stable^38^. Notably, the episomal reprogramming vectors were spontaneously lost in established cPF cell lines (Supplementary Fig. 5a), thus overcoming a current safety concern associated with the integration of viral vector-based reprogramming/conversion approaches. Another potential safety concern regarding cells with proliferative and stem cell capacity is their potential for tumour formation^39^. Transplantation of expanded cPF cells under the kidney capsule of immunodeficient mice did not result in tumour formation even after prolonged periods up to 24 weeks *in vivo* (*n*=10) (Supplementary Fig. 5c). In contrast, all controls (hESC-derived endodermal progenitor cell populations) formed tumorigenic structures with big cysts and increased graft size 7 weeks after transplantation (*n*=4) (Supplementary Fig. 5c). Haematoxylin and eosin staining of cPF grafts showed epithelial structures (Supplementary Fig. 5d) and further immunofluorescence analysis demonstrated protein expression of characteristic endodermal markers, including E-cadherin, HNF4α, PDX1, SOX9 and pan-cytokeratin (Fig. 2j). In summary, our results demonstrate that cPF cells can be greatly expanded in culture while maintaining their posterior foregut endodermal phenotype.

### Characterization of pancreatic endodermal progenitor cells

**Figure 3:**
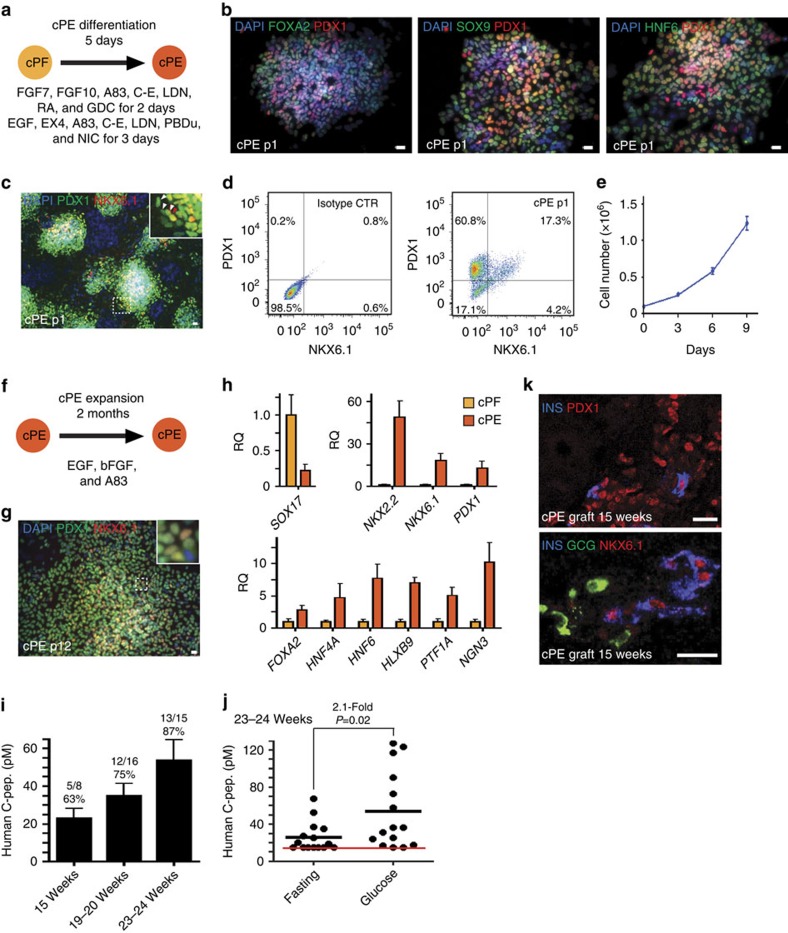
Differentiation of cPF cells into expandable pancreatic endodermal progenitor cells. (**a**) Schematic strategy for the differentiation of cPF cells into pancreatic cPE cells. (**b**,**c**) Immunofluorescence staining of PE markers FOXA2, SOX9, HNF6, PDX1 and NKX6.1 in p1 cPE cells. Scale bar, 20 μm. (**d**) Flow cytometric analysis of PDX1 and NKX6.1 expression in p1 cPE cells. (**e**) Growth curve of cPE cells. Data from three experiments are shown as average±s.e.m. (**f**) Illustration for the expansion of cPE cells. (**g**) Immunofluorescence staining of PE markers PDX1 and NKX6.1 in p12 cPE cells. Scale bar, 20 μm. (**h**) qPCR analysis demonstrated the enrichment of *NKX2.2*, *NKX6.1*, *PDX1, FOXA2, HNF4A, HNF6, HLXB9, PTF1A* and *NGN3*, while *SOX17* is downregulated in cPE cells. Mean values±s.e.m. are normalized to *GAPDH* and relative to cPF cells (*n*=3 experiments). (**i**) ELISA analysis reveals detectable levels of human C-peptide in serum of 67% of mice-bearing cPE cell grafts for 15 weeks 1 h after glucose challenge. Human C-peptide levels and percentage of mice exhibiting detectable levels of human C-peptide increases over time. Numbers on top of each bar indicate human C-peptide-positive mice out of total mice assayed. (**j**) ELISA analysis before and after glucose challenge of mice-bearing cPE cell grafts for 23–24 weeks demonstrates their functional response to glucose administration. Red line indicates values below or equal to detection limit of the ELISA assay. *P* value was calculated using a two-tailed Student's *t*-test. (**k**) Immunofluorescence analysis of 15-week-old cPE cell graft sections shows co-expression of insulin (INS) and the beta-cell transcription factors PDX1 and NKX6.1 but not the hormone glucagon (GCG). Scale bar, 20 μm.

Recently, several studies have reported the use of small molecules and growth factors to achieve differentiation of hESC-derived primitive gut tube and posterior foregut endoderm into pancreatic endoderm^3^,^4^,^11^,^40^. Using a similar approach^7^,^8^, we screened different combinations of small molecules and growth factors to differentiate cPF cells into more committed pancreatic endodermal progenitor cells (cPE cells). We optimized a two-step protocol in which cPF cells were first exposed to FGF7, FGF10, A83-01, Compound-E (an inhibitor of Notch signalling), retinoic acid (RA), GDC-0449 (an antagonist of Sonic hedgehog) and LDN-193189 (an inhibitor of BMP4 signalling) for 2 days (Fig. 3a). Subsequently, differentiating cells were treated with EGF, Exendin-4 (an agonist of glucagon (GCG)-like peptide-1), A83-01, LDN-193189, PBDu (an activator of protein kinase C), Compound-E and Nicotinamide (an inhibitor of polyADP-ribose synthetase) for another 3 days (Fig. 3a). Resulting cPE cells continued to express high levels of FOXA2, HNF6, SOX9 and PDX1, as expected^41^,^42^ (Fig. 3b). Most importantly, this treatment resulted in the generation of PDX1 and NKX6.1 double-positive cells (Fig. 3c). NKX6.1 expression in common PDX1-positive pancreatic progenitors (before NKX6.1 expression becomes further restricted to beta cells) marks their commitment to a more specific endocrine-/ductal-bi-potent progenitor cell type^43^. Notably, hESC-derived PDX1 and NKX6.1 double-positive PE progenitor cells have been shown to be able to give rise to functional beta cells after transplantation^16^. Fluorescence-activated cell sorting (FACS) analysis revealed approximately 78% of PDX1-positive cells, and 17.3% PDX1 and NKX6.1 double-positive cells within cPE populations at passage 1 (Fig. 3d). The level of NKX6.1 expression detected in cPE cells is comparable to PE cells differentiated from hESCs as described by more conventional means (Supplementary Fig. 7a). Next, we tested the cPF expansion media on cPE cultures in an attempt to expand cPE cells in a similar manner. However, cPE colonies easily detached from the plate under cPF expansion condition. Omission of CHIR99021 and increasing the EGF concentration to 50 ng ml^−1^ reversed this effect and promoted cPE cell expansion (Fig. 3f). Employing this optimized culture condition, cPE cells were expanded with an approximate doubling time of 3 days for up to 14 passages (Fig. 3e). Of note, expanded cPE cells maintained their progenitor identity as evidenced by the presence of PDX1 and NKX6.1 double-positive cells at passage 12 (Fig. 3g). Consistently, qPCR results demonstrated the downregulation of early endodermal marker gene *SOX17*. The pan-endodermal marker genes *FOXA2* and *HNF4A*, as well as many pancreatic and endocrine marker genes, including *HNF6*, *PTF1A*, *HLXB9* and *NGN3*, were upregulated (Fig. 3h). More robust expression was noted for other markers, including *PDX1*, *NKX2.2* and *NKX6.1*, which are also highly expressed in mature beta cells.

To explore whether expanded cPE cells can further mature into functional beta cells *in vivo*, we transplanted cPE cells under the kidney capsule of immunodeficient mice. After 15–16, 19–20 and 23–24 weeks, we detected human C-peptide after a glucose challenge in the blood of 62.5%, 75% and 86.6% of mice analysed, respectively, *albeit* at low levels (Fig. 3i). In addition, we detected a 2.1-fold increase in human C-peptide in the serum of mice-bearing 23-week-old grafts after glucose challenge when compared with fasting levels, illustrating that cPE grafts gave rise to functional beta-like cells on transplantation into host animals (Fig. 3j). Starting at 15 weeks post transplantation, we found insulin-expressing beta-like cells and PDX1-positive pancreatic progenitor cells in some graft sections, while other regions were negative for pancreatic markers (Fig. 3k). Insulin-positive cells co-expressed the critical beta-cell transcription factors PDX1 and NKX6.1, but did not show expression of other endocrine hormones (Fig. 3k). In addition, we detected CK19-positive duct-like structures, but not Amylase-positive exocrine cells, a finding that supports the notion that cPE cells possess bi-potential duct/endocrine progenitor capacity (Supplementary Fig. 7b). In summary, our data demonstrates that cPF cells can be differentiated into cPE cells, which can be greatly expanded *in vitro* while maintaining their specific phenotype. Importantly, cPE cells differentiate further *in vivo* towards insulin-producing, single-hormonal cells capable of releasing insulin in response to glucose challenge.

### Maturation of cPE cells into pancreatic beta-like cells

**Figure 4:**
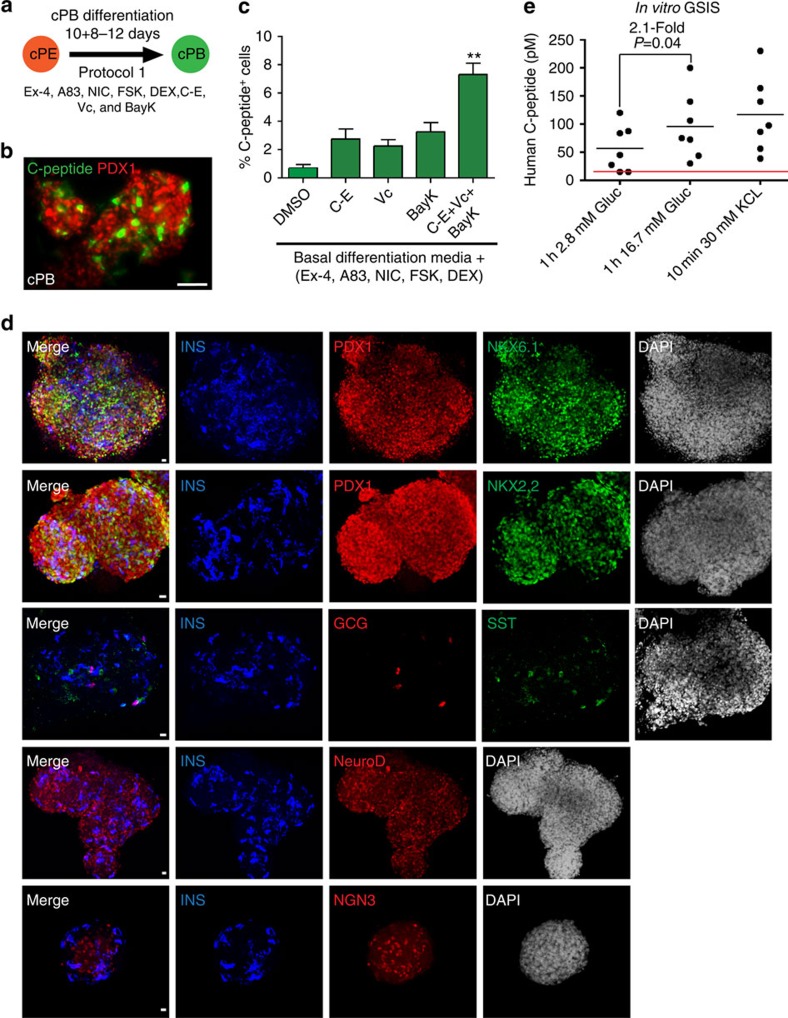
Maturation of cPE cells into insulin-producing, glucose-responsive pancreatic beta-like cells *in vitro*. (**a**) Schematic representation of protocol 1 differentiation strategy employed to mature cPE cells into pancreatic beta-like cells (cPB cells) *in vitro*. (**b**) Immunofluorescence staining of PDX1 and C-peptide expression in beta-like cells generated with basal pancreatic differentiation condition *in vitro*. Scale bar, 20 μm. Basal pancreatic differentiation media contains A83-01 (A83), Nicotinamide (NIC), Forskolin (FSK), Dexamethasone (DEX) and Exendin-4 (Ex-4). (**c**) Several small molecules, Compound-E (C–E), Vitamin C (Vc) and BayK-8644 (BayK) further increase the percentage of C-peptide-positive cells. Note that combined treatment of all molecules results in an additive effect, further increasing the percentage of C-peptide-positive cells (*n*=3 experiments). Statistical significance calculated using two-tailed Student's *t*-test, compared with DMSO controls. **P<0.01. (**d**) Immunofluorescence analysis of converted pancreatic beta-like cells (cPB cells) generated with the improved pancreatic maturation conditions. Many of the insulin (INS) positive cells co-express key beta-cell transcription factors including, PDX1, NKX6.1, NKX2.2 and NEUROD1, but only rarely co-express endocrine progenitor marker NGN3 and the endocrine hormones, glucagon (GCG) and somatostatin (SST). Scale bar, 20 μm. (**e**) *In vitro*, glucose-stimulated insulin secretion (GSIS) assays (*n*=7 cell cultures of 4 experiments) demonstrated that cPB cells release insulin in response to physiological levels of glucose. Depolarization by higher KCl concentration further increased insulin secretion. Note that insulin release was measured by human-specific C-peptide ELISA assay. Red line indicates values below or equal to detection limit of the ELISA assay. *P* value was calculated using a two-tailed Student's *t*-test.

To address the question of whether cPE cells can develop into functional beta-like cells under cell culture conditions, we first incubated cPE cells in a basal pancreatic differentiation media that has been shown to promote hESC/iPSC-derived pancreatic progenitor differentiation into insulin-producing cells^40^. This media includes A83-01, Nicotinamide, Forskolin (an activator of adenylyl cyclase), Dexamethasone (an agonist of glucocorticoid receptor) and Exendin-4 (Fig. 4a). While we consistently observed C-peptide-positive cells expressing high levels of PDX1 after 10–14 days in culture (Fig. 4b), the relatively low number (∼0.5%) suggested that further optimization was required. Chemical compounds able to promote differentiation of definitive endoderm^44^ and PDX1-positive progenitors^9^ have been identified previously. However, discovering molecules directing the final steps of differentiation into beta-like cells has been challenging due to difficulties in generating and maintaining sufficient numbers of differentiation-competent PE cells. We performed a chemical screen^45^ with the intent of identifying factors that would result in more efficient development of C-peptide-positive cells from expanded cPE cells. These experiments revealed that supplementation of the basal pancreatic differentiation media individually with Compound-E (an inhibitor of Notch signalling), Vitamin C or BayK-8644 (a Ca^2+^ channel agonist) were effective in increasing the percentage of C-peptide-positive cells (Fig. 4c). Combined treatment with these compounds had an additive effect (Exendin-4, A83-01, Nicotinamide, Forskolin, Dexamethasone, Compound-E, Vitamin C and BayK-8644), resulting in the formation of up to 7% C-peptide-positive cells (Fig. 4c).

Previously, insulin-producing cells generated from ESCs/iPSCs are mostly immature as evidenced by co-expression of endocrine hormones, the lack of crucial beta-cell transcription factors and the inability to secrete insulin in response to physiological levels of glucose^46^. The maturation defects may be partly due to the lack of three-dimensional (3D) organization normally present in islets of Langerhans^47^. Hence, we generated free-floating cell aggregates of cPE cells that first had been incubated as 2D cultures for 10 days in the improved maturation media (protocol 1). The majority of cells within the aggregates expressed the pan-pancreas marker PDX1 after 8–12 days of 3D culture (Fig. 4d; Supplementary Fig. 8a,b). In addition, we detected many insulin-expressing cells co-expressing key beta-cell transcription factors, including PDX1, NKX6.1, NKX2.2 and NEUROD1 (Fig. 4d; Supplementary Fig. 8b). In contrast, insulin-expressing cells only rarely co-stained for the endocrine progenitor marker NGN3 or other endocrine hormones, including GCG and somatostatin (SST) (Fig. 4d; Supplementary Fig. 8b). Of note, all insulin-positive cells also stained for a human-specific C-peptide antibody, thus excluding possible insulin uptake from the media (Supplementary Fig. 8c). Considering the expression of key beta-cell markers, we designate these cells as converted pancreatic beta-like cells (cPB cells). One of the distinguishing hallmarks of pancreatic beta cells is the ability to release insulin upon glucose stimulation. Importantly, glucose-stimulated insulin secretion (GSIS) assays demonstrated that *in vitro*-generated cPB cells released insulin in response to physiological levels of glucose (*n*=7, fold increase 2.1±1.3) (Fig. 4e). Collectively, our findings illustrate the generation of functional pancreatic beta-like cells that respond to physiological levels of glucose *in vitro*.

### cPB cells protect mice from STZ-induced diabetes

**Figure 5:**
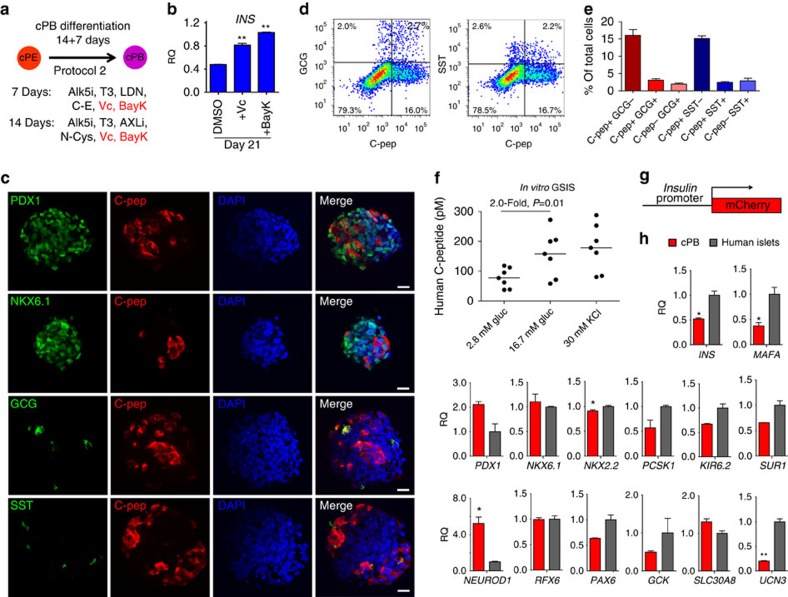
Improved maturation of cPE cells into insulin-producing, glucose-responsive cPB cells *in vitro*. (**a**) Schematic representation of the improved approach (protocol 2) employed to mature cPE cells into cPB cells *in vitro*. The improved protocol 2 consists of two candidate factors, Vitamin C (Vc) and BayK-8644 (BayK), identified by our chemical screen in conjugation with recently published protocols^7^,^8^. (**b**) Addition of Vitamin C (Vc) and BayK-8644 (BayK) increases mRNA levels of *INSULIN (INS)* gene in differentiated cPB cultures at day 21. *n*=3 experiments. Statistical significance calculated using two-tailed Student's *t*-test, compared with DMSO controls. **P<0.01. (**c**) Immunofluorescence analysis of cPB cells generated with the improved pancreatic maturation conditions. Many of the C-peptide (C-pep)-positive cells co-express key beta-cell transcription factors, PDX1 and NKX6.1, but only rarely co-express other endocrine hormones, glucagon (GCG) and somatostatin (SST). Scale bar, 50 μm. (**d**) Flow cytometric analysis of cPB cells for human C-peptide (C-pep), glucagon (GCG) and somatostatin (SST). (**e**) Quantification of flow-based analysis of the percentage of single- and double-positive cells for C-pep and GCG or SST. *n*=3 experiments. (**f**) *In vitro*, glucose-stimulated insulin secretion (GSIS) assay (*n*=7 cell cultures of 3 experiments) demonstrate that cPB cells release insulin in response to physiological levels of glucose. Depolarization by higher KCl concentration further increased insulin secretion. Note that insulin release was measured by human-specific C-peptide ELISA assay. *P* value was calculated using a two-tailed Student's *t*-test. (**g**) Schematic of the lentiviral reporter construct employed to infect cPE cultures before differentiation into cPB cells. (**h**) Insulin-expressing cPB cells at day 21 were sorted based on mCherry expression. qPCR analysis of sorted cPB cells in comparison to primary human islets shows comparable expression levels of key beta-cell genes, including *INS*, *PDX1*, *NKX6.1*, *NKX2.2*, *NEUROD1*, *PAX6*, *RFX6*, *MAFA*, *GCK*, *PCSK1*, *KIR6.2*, *SUR1*, *UCN3* and *SLC30A8. P* value was calculated using a two-tailed Student's *t*-test. *P<0.05, **P<0.01.

Functional insulin-producing cells are capable of regulating glucose levels in surrogate animals. Unfortunately, our initial trials in which cPB cells (protocol 1) were transplanted into immunocompromised, streptozotocine (STZ)-treated diabetic mice were unsuccessful, with most animals retaining high glucose levels and eventually succumbing to diabetes without recovery. Encouraged by two recent papers that introduced improved protocols for pancreatic differentiation^7^,^8^, we further improved the maturation conditions of cPE cells into cPB cells (protocol 2) (Fig. 5a). In addition to the Notch inhibitor that we already employed in protocol 1 and that was also included in both published protocols, we tested whether the addition of Vitamin C and BayK-8644, which were discovered during our initial chemical screens, had additional beneficial effects. We found that both compounds promoted expression of the *INSULIN* gene (Fig. 5b) as well as increased the number of C-peptide-positive cells co-expressing key beta-cell transcription factors PDX1 and NKX6.1 (Fig. 5c). C-peptide-positive cells rarely co-stained for other endocrine hormones, including GCG and SST (Fig. 5c). Quantitative FACS analysis revealed the formation of about 15% monohormonal C-pep^+^GCG^-^SST^-^ cells, with only few polyhormonal cells (Figs 5d,e). We infected cPE cells with a lentivirus containing a minimal *insulin* promoter driving a fluorescence reporter expression and FACS-sorted cPB cells at d21 (Fig. 5g). qPCR analysis for the expression of beta-cell marker genes, including *INS*, *PDX1*, *NKX6.1*, *NKX2.2*, *NEUROD1*, *PAX6*, *RFX6*, *MAFA*, *GCK*, *PCSK1*, *KIR6.2*, *SUR1*, *UCN3* and *SLC30A8* of sorted cPB cells in comparison to human islets showed comparable levels for most genes, with only few genes showing slightly reduced levels of expression (Fig. 5h).

GSIS assays conducted with *in vitro*-generated cPB cells revealed a robust release of insulin in response to physiological levels of glucose (*n*=7, fold increase 2.0±0.4) (Fig. 5f; Supplementary Fig. 9a). The stimulation index, as calculated by the ratio of insulin secreted in high glucose to low glucose, was similar among the cPB cells, hESC-derived beta-like cells and primary human islets^6^,^7^,^8^. Thus, our findings illustrate that protocol 2 efficiently generates functional pancreatic beta-like cells *in vitro*.

**Figure 6:**
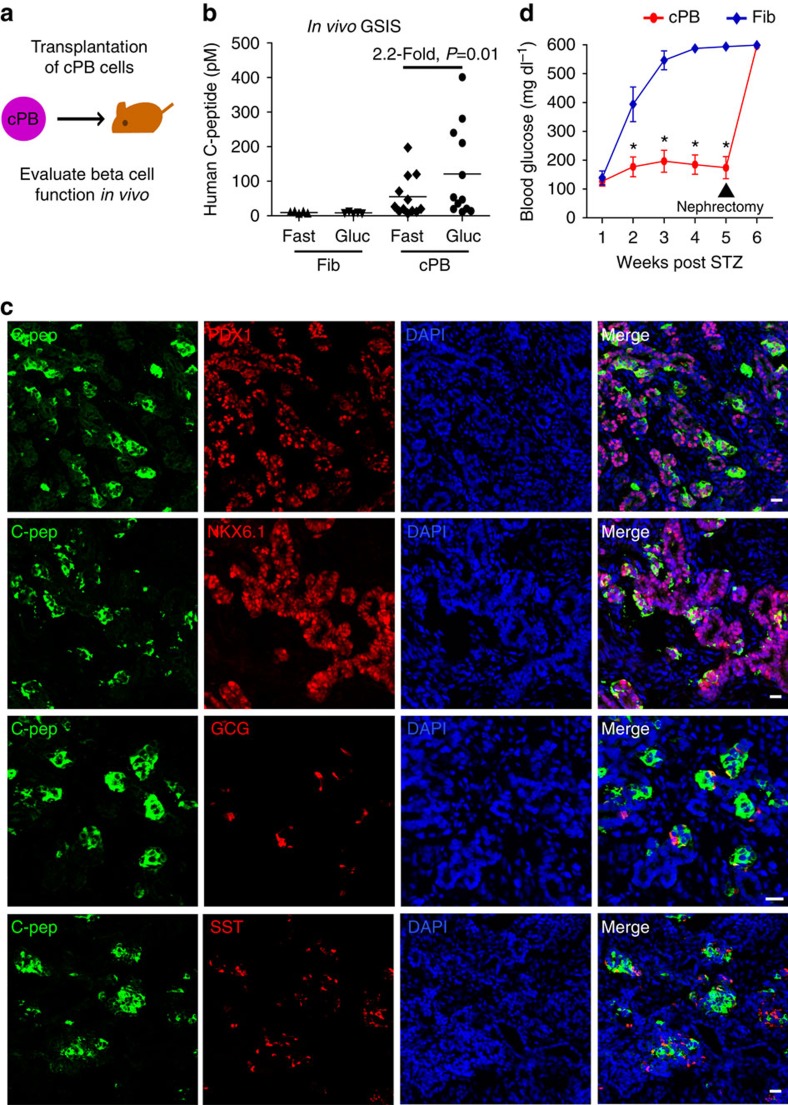
Transplanted cPB cells remain functional and protect mice from chemically induced diabetes. (**a**) Schematic representation of the transplantation of cPB into immunodeficient mice. (**b**) ELISA analysis of serum from fasted and glucose-challenged mice 2 months post transplantation with either fibroblasts (Fib) or cPB are shown. cPB graft-bearing mice exhibit significant higher levels of circulating human C-peptide in serum after a glucose bolus, indicating that transplanted cPB cells remain functional *in vivo*. Mice transplanted with Fib controls do not exhibit circulating human C-peptide. *n*=12 mice for cPB and *n*=5 mice for Fib. *P* value was calculated using a two-tailed Student's *t*-test. (**c**) Immunofluorescence analysis of 2-month-old cPB cell grafts shows co-expression of C-peptide (C-pep) and the beta-cell transcription factors PDX1 and NKX6.1 but not the hormones glucagon (GCG) and somatostatin (SST). Scale bar, 50 μm. Data shown are representative of two mice. (**d**) Fed blood glucose levels of mice-bearing cPB grafts with circulating human C-peptide levels above 200 pM after glucose stimulation and control fibroblasts are shown. Mice were treated with the mouse-specific beta-cell toxin streptozotocine (STZ) to ablate endogenous beta cells. Uni-lateral nephrectomy of cPB graft-bearing mice 5 weeks after STZ treatment resulted in a rapid rise in blood glucose levels, directly demonstrating euglycemic control due to cPB grafts after STZ treatment in these mice. *n*=6 mice for cPB and *n*=6 mice for Fib. *P* value was calculated using a two-tailed Student's *t*-test. *P<0.05.

Considering these major improvements for *in vitro*-generated cPBs, we decided to re-evaluate their *in vivo* function by transplanting cPB cells into immunodeficient mice (Fig. 6a). Importantly, while control human fibroblasts did not evoke any response, we detected significant glucose-stimulated insulin secretion in cPB-transplanted mice 2 months after transplantation (Fig. 6b; Supplementary Fig. 9b). Immunofluorescence analysis of cPB grafts revealed the presence of many islet-like structures containing insulin-expressing pancreatic beta-like cells. C-peptide-positive cells co-expressed critical beta-cell transcription factors PDX1 and NKX6.1, but did not exhibit expression of other endocrine hormones GCG and SST (Fig. 6c). Specific ablation of the endogenous beta cells by the toxin STZ resulted in rapid onset of overt diabetes in all control animals, while cPB-bearing mice remained euglycemic (Fig. 6d). 5 weeks post-STZ treatment, cPB grafts were removed by uni-lateral nephrectomy. Nephrectomized mice rapidly developed diabetes, demonstrating that human fibroblast-derived cPB cells controlled glucose levels in STZ-treated mice. In summary, these results directly demonstrate that cPB cells transplanted into surrogate mice are functional *in vivo* and protect mice from diabetes.

### Generating pancreatic beta-like cells from adult human fibroblasts

We applied our pancreatic conversion strategy to adult human fibroblasts and established six cPF cell lines from three independent experiments that were further expanded. Using the same culture conditions as for neonatal fibroblast-derived progenitors, we were able to generate expandable cPF and cPE cells from adult fibroblasts (Supplementary Fig. 10a,b). We checked the genome stability of cPF cells using comparative genomic hybridization arrays and did not observe any gross chromosomal aberrations, but only one copy number variant in one adult fibroblast-derived cPF cell line (Supplementary Fig. 11), indicating their genome stability. cPE cells could further differentiate into pancreatic beta-like cells at efficiencies comparable to neonatal fibroblast-derived cPB cells, with only few double hormone-positive cells (Supplementary Fig. 12a–c). Most importantly, human adult fibroblast-derived cPB cells are functional as demonstrated by their glucose-stimulated insulin secretion (Supplementary Fig. 12d). While results from the analysis of neonatal and adult fibroblast-derived pancreatic cells are remarkably similar, we do acknowledge certain batch-to-batch variation among experiments; a finding that mirrors prior reports observing variations among primary adult beta-cell preparations and stem cell-derived beta-like cells^6^,^7^,^8^. Therefore, our results demonstrate that human adult fibroblasts can be used to efficiently and rapidly generate functional cPB cells, a finding that opens up opportunities for patient-specific analysis of beta-cell properties and cell therapy approaches.

## Discussion

Various strategies towards the generation of functional beta cells are currently being pursued, including directed differentiation of pluripotent stem cells^2^,^3^,^4^,^6^,^7^,^8^ and direct reprogramming of different endodermal cell types^48^,^49^,^50^,^51^. In this study, we (I) developed a simple, robust and non-integrating approach for the inter-lineage conversion of mesodermal human fibroblasts into definitive endodermal progenitor cells (cDE cells) following the CASD transdifferentiation approach; (II) showed that cDE cells could be further specified into posterior foregut-like progenitor (cPF) cells that can be greatly expanded; (III) demonstrated that cPF cells can be further differentiated into expandable pancreatic endodermal progenitor (cPE) cells; (IV) reported that these cPE cells can give rise to functional pancreatic beta-like (cPB) cells *in vitro*; (V) and demonstrated that transplanted cPB cells exhibit glucose-stimulated insulin secretion *in vivo* and protect mice from chemically induced diabetes.

By following the CASD transdifferentiation paradigm^17^,^18^,^21^, we show that conversion of fibroblasts into proliferative progenitor populations with the ability to give rise to beta cells represents an attractive and feasible approach for the generation of vast numbers of human beta cells under cell culture conditions. The current efficiency of the conversion process is low and we expect higher yields on future improvements similar to those previously accomplished for iPSC reprogramming^45^. While the approach is robust, the individual roles of reprogramming factors as ‘pioneer transcription factors' and the detailed analysis of the underlying molecular mechanisms of the conversion process require more systematic investigations^52^,^53^,^54^. Employing transient expression of iPSC- reprogramming factors^31^ under the CASD approach, likely establishes a permissive state in cells, that can be exploited by incubation in culture conditions previously shown to favour definitive endoderm derivation^32^,^33^,^34^. This notion is supported by the observation that addition of epigenetic chemical modulators significantly increased the conversion efficiency.

Notably, the resulting cDE cells could rapidly be specified towards the posterior foregut state (cPF), suggesting efficient transdifferentiation of the fibroblasts towards the endodermal germ layer. Subsequently, we established cPF cell lines that can be greatly expanded without loss of their specific identity. This kind of virtually unlimited expansion is reminiscent of the recently described generation and expansion of endodermal progenitor cells from hESCs (eEP)^14^. However, eEP cells have been described as definitive endodermal progenitors while our established cPF cells resemble a further specified posterior foregut-like cell type. The difference in developmental stage might be explained by the different expansion conditions for cPF cells, relying solely on activation of the WNT pathway and inhibition of the TGFβ pathway in combination with pro-proliferative growth factors EGF and bFGF. A somewhat unexpected finding was the observation that we can expand not only cPF cells, but also cPE cells committed towards a pancreatic phenotype. The expansion of cPE cells by at least two hundred million fold involved the addition of a selective set of factors, including bFGF, EGF and compound A83-01. A role for EGF in short-term proliferation of hESC-derived PE cells has been suggested previously^2^,^55^. Interestingly, we found that inhibition of the TGFβ pathway by A83-01 was particularly important for the expansion process of cPE cells, a finding that correlates well with prior reports showing that inhibition of TGFβ signalling is beneficial for pancreatic specification of differentiating hESCs^11^. Mechanistically, inhibition of TGFβ signalling might promote epithelial cell proliferation while at the same time interfering with the commonly observed culture induced epithelial-to-mesenchymal transition process^56^. It would be particularly interesting to test whether the cPE expansion conditions we identified can also be employed to amplify hESC-derived PE cells in a similar manner. Future in-depth analysis of our endodermal progenitor populations and their comparison with progenitor cells generated by distinct methods, such as eEP cells or hESC-derived PE cells, will provide invaluable information about the mechanisms underlying endodermal progenitor specification and self-renewal in a human setting that has remained hard to study due to the limited supply of embryonic samples at the relevant stages.

Importantly, employing a chemical library screen on expanded cPE cells *in vitro* resulted in the discovery of several small molecules, including the Notch inhibitor Compound-E, Vitamin C and the Ca^2+^ channel agonist BayK-8644 that could improve the differentiation efficiency into pancreatic beta-like cells independently as well as in combination. Notch inhibition in bi-potent PE cells induces expression of NGN3, a factor essential for endocrine cell development^57^,^58^, and similar findings have recently been reported for conventional hESC to beta-cell differentiation protocols^7^,^8^. The effects of Vitamin C and BayK-8644 on beta-cell differentiation have been less well-studied. Of note, our group described previously that Vitamin C was effective in promoting mouse pancreatic beta-cell differentiation and maturation^30^. Recent reports suggested Vitamin C is involved in many epigenetic modifications, including DNA and histone demethylation^59^, promotes cell fate conversion^60^ and improves cell quality^61^,^62^. Therefore, future studies will explore the roles of Vitamin C and related epigenetic regulators in pancreatic differentiation and maturation^63^,^64^,^65^. With regard to the functional properties of BayK-8644, one very attractive underlying mechanism could be related to the Ca^2+^/Calcineurin (Cn)/Nuclear Factor-Activated T cells (NFAT) signalling pathway, known to promote mouse and human pancreatic beta-cell proliferation and maturation^66^,^67^,^68^. More detailed analysis of the mechanistic functions of these compounds in future studies might reveal novel properties of pancreatic beta-cell differentiation and function that could be exploited for cell therapy approaches.

Significantly, our *in vitro*-differentiated beta-like cells exhibit a functional beta-cell phenotype as judged by their ability to secrete insulin in response to physiological levels of glucose, as well as their co-expression of critical beta-cell transcription factors. While we observed a similar fold change in human C-peptide secretion on glucose stimulation of cPB cells in comparison to recently described hESC-derived beta-like cells and human islets, we note a wider range of secretion, similar to what has been reported for iPSC-derived beta-like cells^7^,^8^. Further, in-depth analysis may reveal additional genes and epigenetic modifiers that regulate the maturation process of pancreatic beta cells from somatic cells. Nonetheless, even in its present state our findings clearly indicate the potential of human adult fibroblast-derived functional beta-like cells for the identification and analysis of novel patient-specific properties of these insulin-producing cells.

Finally, the transplantation experiments of cPB cells into immunodeficient mice highlight their ability to give rise to functional islet-like structures in this physiological context. This is in contrast to human iPSC-derived pancreatic beta-like cells which, despite their ability to respond to glucose-stimulated insulin secretion *in vitro* and *in vivo*, have not been reported to be able to protect immunocompromised mice from STZ-induced diabetes on transplantation^7^,^8^. Thus, our studies represent one of the few examples of human cell types generated through cellular reprogramming that could protect against or even cure an existing disease^19^.

We are cognizant of the fact that our cPB cell cultures do not contain all cells typically found in human islets, including mesenchymal support cells, endothelial cells, and neural cells. Thus, even while we observe clear evidence for the functional properties of the fibroblast-derived beta-like cells, we anticipate that future co-culture experiments aimed at fully replicating the cellular composition of human islets will further optimize the function of cPB cells. Furthermore, there is likely an *in vivo* maturation process of *in vitro*-engineered pancreatic cells, as reported in recent studies of hESC-derived pancreatic beta cells^7^,^8^, whose underlying mechanisms have not been fully elucidated yet. We anticipate that understanding the signals that guide beta-cell maturation will allow generating human fibroblast-derived cells fully recapitulating all properties of human pancreatic beta cells.

In summary, our study demonstrates an effective conversion strategy for human fibroblasts into the endodermal lineage, describes robust expansion conditions for distinct endodermal progenitor cell populations and identifies novel cocktails of signalling factors that promote the successful differentiation of these endodermal progenitor cells into functional beta-like cells both *in vitro* and *in vivo*. Most significantly, when transplanted into immunocompromised mice, these cPB cells produced human insulin and protected mice from diabetes. We surmise that the current CASD strategy is a viable alternative to commonly used iPSC approach and that these efforts will advance our understanding of basic pancreas biology and disease modelling, as well as the development of cell replacement therapies and drug testing strategies.

## Methods

### Conversion of human fibroblasts into endodermal progenitor cells

Human foreskin fibroblasts (CRL-2097, ATCC) and adult human dermal fibroblasts (Cell Applications) were cultured in a 10-cm tissue-culture dish in regular fibroblast culture medium. Reprogramming with episomal vectors was done as described^31^. Briefly, 4 × 10^5^ fibroblasts were electroporated with up to 6 μg of episomal vectors (pCXLE-hOCT3/4-shp53-F, pCXLE-hSK and pCXLE-EGFP) using the Microporator Human Dermal Fibroblast (NHDF) Nucleofector Kit (Lonza) according to the manufacturer's instructions. Cells were cultured in fibroblast medium for 4 days and then re-plated onto Matrigel-coated 10-cm dishes at a density of 50 000 cells per dish. The cells were then cultured in reprogramming initiation medium (DMEM/F12, 10% Knockout serum replacement, 5% ES-FBS, 1% Glutamax, 1% Non-essential amino acids, 1% penicillin/streptomycin, 0.1 mM β-mercaptoethanol, 10 ng ml^−1^ bFGF, 10 ng ml^−1^ EGF, 2 μM Par, 0.5 μM RG, 0.1 mM NaB, 0.5 μM NECA and 3 μM CHIR99021) for 1 week, followed by endodermal induction medium (Advanced RPMI, 2% ES-FBS, 1% Glutamax, 1% Non-essential amino acids, 1% penicillin/streptomycin, 0.1 mM β-mercaptoethanol, 2 μM Par, 0.5 μM RG, 0.1 mM NaB, 0.5 μM NECA, 3 μM CHIR99021 and 100 ng ml^−1^ Activin A) for another 2–3 weeks. The converted colonies were carefully picked up for expansion in expansion medium (DMEM, 1% Glutamax, 0.5 × N2, 0.5 × B27, 5 μg ml^−1^ BSA, 1% penicillin/streptomycin, 10 ng ml^−1^ bFGF, 10 ng ml^−1^ EGF, 0.5 μM A83-01 and 3 μM CHIR99021) and were passaged at 1:4–1:6 each time by Accutase treatment. Routinely, 0.5 μM thiazovivin was used during the first 12–24-h period of each passage to prevent cell death after dissociation. All cell culture products were from Invitrogen/Gibco BRL and all chemicals and growth factors were from Stemgent except where mentioned.

### Differentiation of cPF cells into cPE cells

For pancreatic differentiation, cPF cells were cultured in pancreatic differentiation medium (DMEM, 1% Glutamax, B27, 5 μg ml^−1^ BSA, 1% penicillin/streptomycin) with 25 ng ml^−1^ FGF7, 25 ng ml^−1^ FGF10, 0.5 μM A83-01, 0.1 μM Compound-E, 2 μM RA, 0.1 μM GDC-0449 and 0.1 μM LDN-193189 for 2 days; then 50 ng ml^−1^ EGF, 50 ng ml^−1^ Exendin-4, 0.5 μM A83-01, 0.1 μM Compound-E, 50 nM TPB, 0.1 μM LDN-193189 and 10 mM nicotinamide for another 3 days. After differentiation, the cPE cell population was passaged with Accutase and cultured in cPE expansion medium (DMEM, 1% Glutamax, 1 × B27, 5 μg ml^−1^ BSA, 1% penicillin/streptomycin, 10 ng ml^−1^ bFGF, 50 ng ml^−1^ EGF and 0.5 μM A83-01). Routinely, 0.5 μM thiazovivin was used during the first 12–24 h period of each passage to prevent cell death after dissociation.

### Maturation of cPE cells into cPB cells

*Protocol 1*. The cPE cells were differentiated into beta-like cells in the pancreatic maturation media with DMEM, 1% Glutamax, B27, 5 μg ml^−1^ BSA and 1% penicillin/streptomycin, 50 ng ml^−1^ Exendin-4, 1 μM A83-01, 10 μM forskolin, 10 μM dexamethasone, 10 mM nicotinamide, 0.1 μM Compound-E, 50 μg ml^−1^ Vitamin C and 2 μM BayK-8644 for 10 days, and then cultured as 3D aggregates in low-attachment plates for another 8–12 days.

*Protocol 2*. The cPE cells were differentiated into beta-like cells in the pancreatic maturation media with DMEM, 1% Glutamax, B27, 5 μg ml^−1^ BSA and 1% penicillin/streptomycin, 10 μg ml^−1^ Heparin, 10 μM ZnSO4, 10 μM Alk5 inhibitor II, 0.1 μM LDN-193189, 1 μM T3, 0.1 μM Compound-E, 2 μM BayK-8644, 0.25 mM Vc for 7d; and DMEM, 1% Glutamax, B27, 5 μg ml^−1^ BSA and 1% penicillin/streptomycin, 10 μg ml^−1^ Heparin, 10 μM ZnSO4, 10 μM Alk5 inhibitor II, 1 μM T3, 1 mM N-cysteine, 10 μM Trolox, 2 μM R428, 2 μM BayK-8644 and 0.25 mM Vc for 7 days, and then cultured as 3D aggregates in low-attachment plates for another 7 days.

### Immunofluorescence staining

Standard immunostaining was carried out as previously reported^69^. For cell cluster staining, primary and secondary antibodies were incubated overnight at 4 °C. Secondary antibodies were Alexa Fluor-conjugated (1:500–1,000) (Invitrogen). Nuclei were visualized by Hoechst (Sigma-Aldrich) staining. Images were captured using a Nikon Eclipse TE2000-U microscope or a SP5 confocal microscope. The primary antibodies used are detailed in Supplementary Table 1. A representative picture of at least five independent experiments is shown in Figs 1b,c and 2g,h. A representative picture of at least three independent experiments is shown in Figs 2c,j, 3b,c,g, 4b and 5c and Supplementary Figs 2–4. A representative picture of three independent experiments is shown in Supplementary Figs 1a, 5b, 8b,c, 10a,b and 12a. A representative image of two independent mice is shown in Figs 3k and 6c and Supplementary Fig. 7b.

### Flow cytometry

Cells were harvested at the indicated time points by Accutase treatment, fixed with 4% formaldehyde solution, and washed five times with ice-cold Perm/Wash buffer (BD). Cells were aliquoted and incubated individually or combinatory with antibodies and isotype controls on ice for 2 h. Cells were washed with Perm/Wash buffer for five times and incubated individually with Alexa Fluor 488-conjugated or Alexa Fluor 555-conjugated antibodies (1:500, Invitrogen) on ice for 1 h. Cells were washed with Perm/Wash buffer for five times, re-suspended in 0.5 ml ice-cold PBS with 2% FBS and analysed by FACSCalibur and CellQuest software (BD). FlowJo software (Tree Star) was used to analyse the data.

### QPCR

For qPCR analysis, total RNA was extracted using the RNeasy Plus Mini Kit in combination with QIAshredder (Qiagen). First strand reverse transcription was performed with 1 μg RNA using iScript cDNA Synthesis Kit (Bio-Rad). qPCR was taken out using iQ SYBR Green Supermix (Bio-Rad). The primers used are detailed in Supplementary Table 2.

### Kidney capsule transplantation

Mice (NOD.Cg-Prkdc^scid^ Il2rg^tm1Wjl^/SzJ mice, NSG mice, The Jackson Laboratory) used in this study were maintained according to protocols approved by the University of California, San Francisco, Committee on Laboratory Animal Resource Center. The kidney capsule transplantation was done as previously reported^70^. Briefly, cells were collected from culture dishes by cell scraper, and injected under the renal capsule of immunodeficient NSG male mice (6–10-week-old). For transplantation assays of established cPF cell lines, hESC-derived definitive endoderm generated by differentiation for 1 day in RPMI containing 100 ng ml^−1^ Activin A and 50 ng ml^−1^ WNT3a, followed by 4 days in RPMI containing 0.2% FBS and 100 ng ml^−1^ Activin A served as a control. Grafts were dissected and analysed at the indicated time points. For *in vivo* functional assays of cPB cells, ∼5.0 × 10^6^ cPB cells and control human fibroblasts were transplanted. For glucose-induced insulin secretion, mice were fasted overnight and serum was collected before and after i.p. administration of 3 g kg^−1^ D-glucose solution. For induction of diabetes, mice were administered 35 mg kg^−1^ Streptozotocin via i.p. injection for 5 days.

### Glucose-stimulated insulin secretion

Cell were pre-incubated for 1 h in Krebs–Ringer buffer (KRB), followed by incubation for 1 h in KBR containing 2.8 mM glucose followed by 1 h incubation in KRB containing 16.7 mM glucose followed by 30 min in KRB containing 16.7 mM glucose and 30 mM KCl. Human C-peptide levels were quantified using an ultrasensitive ELISA kit (Mercodia; cross-reactivity with insulin and pro-insulin, 0.0006% and 1.8%, respectively).

### Statistics

Indicated *P* values were obtained using a two-tailed *t*-test, and all quantitative data are shown as mean±s.e.m. No statistical method was used to predetermine sample size. No samples were excluded. The experiments were not randomized. The investigators were not blinded to allocation during the experiments and outcome assessment.

## Additional information

**How to cite this article:** Zhu, S. *et al*. Human pancreatic beta-like cells converted from fibroblasts. *Nat. Commun.* 7:10080 doi: 10.1038/ncomms10080 (2016).

## Supplementary Material

Supplementary Information Supplementary Figures 1-12 and Supplementary Tables 1-2.
