# Supplementary material for: Human pancreatic beta-like cells converted from fibroblasts
Source: Nat Commun. 2016 Jan 6;7:10080. doi: 10.1038/ncomms10080 (PMC4729817; doi:10.1038/ncomms10080)
Supplement: Supplementary Information — Supplementary Figures 1-12 and Supplementary Tables 1-2. [file ncomms10080-s1.pdf]

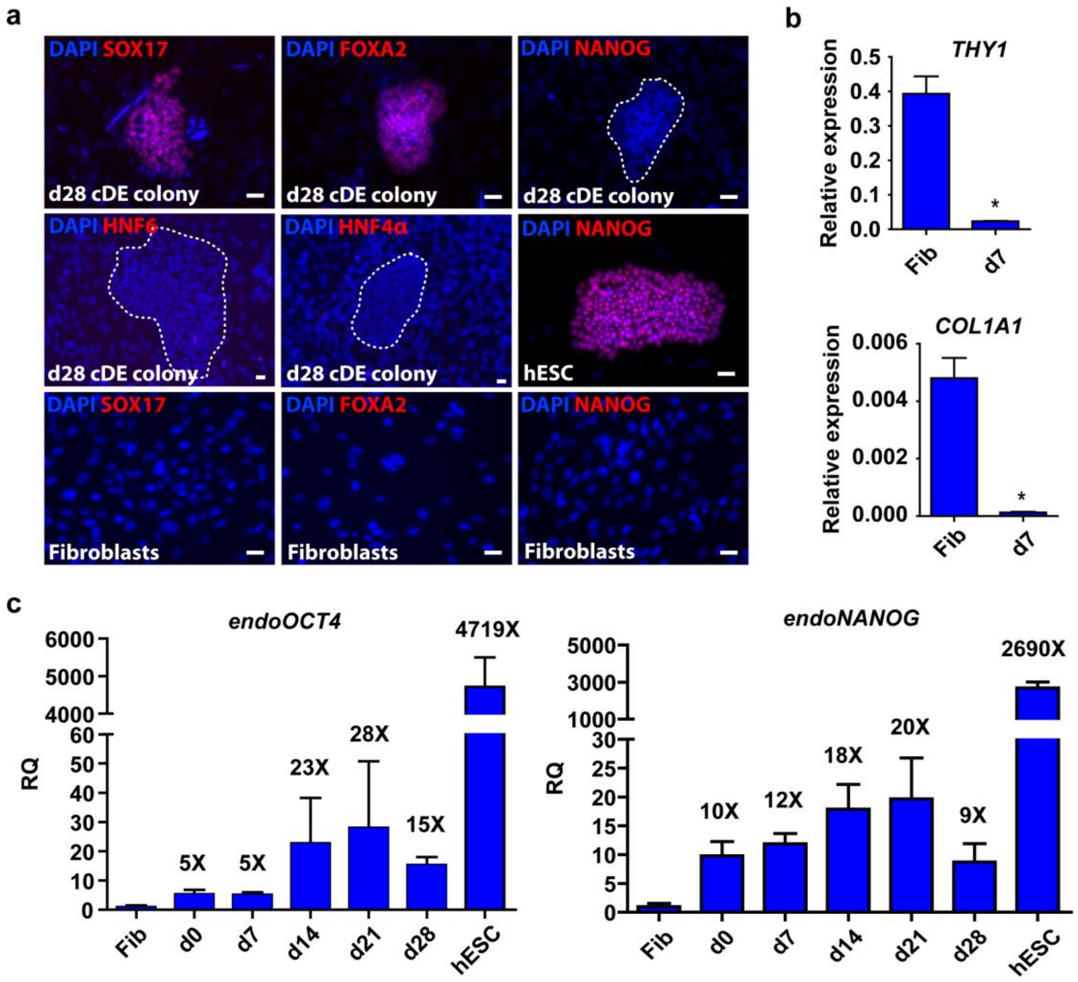

Supplementary Figure 1. Immunofluorescence analysis of endodermal and pluripotency markers in definitive endodermal progenitors converted from fibroblasts. (a) cDE colonies at day 28 express early endodermal progenitor markers SOX17 and FOXA2, but not primitive gut tube marker HNF4α, posterior foregut marker HNF6, and pluripotency marker NANOG. Note that hESCs served as positive control for NANOG staining. Control fibroblasts do not express any marker analyzed. Scale bar, 20 μm. **(b)** qPCR analysis revealed marked downregulation of transcript levels of two fibroblasts marker genes, *THY1* and *COL1A1*, 7 days after initiating conversion. **(c)** qPCR analyses of expression levels of endogenous pluripotent genes *OCT4* (endo*OCT4*) and *NANOG* (endo*NANOG*) during the conversion process. Note that fibroblasts (Fib) and hESCs served as negative and positive control, respectively. Mean values ± s.e.m. are normalized to *Glyceraldehyde 3-phosphate dehydrogenase* (*GAPDH*) and relative to Fib (n=3 experiments). Fold changes relative to Fib are shown.

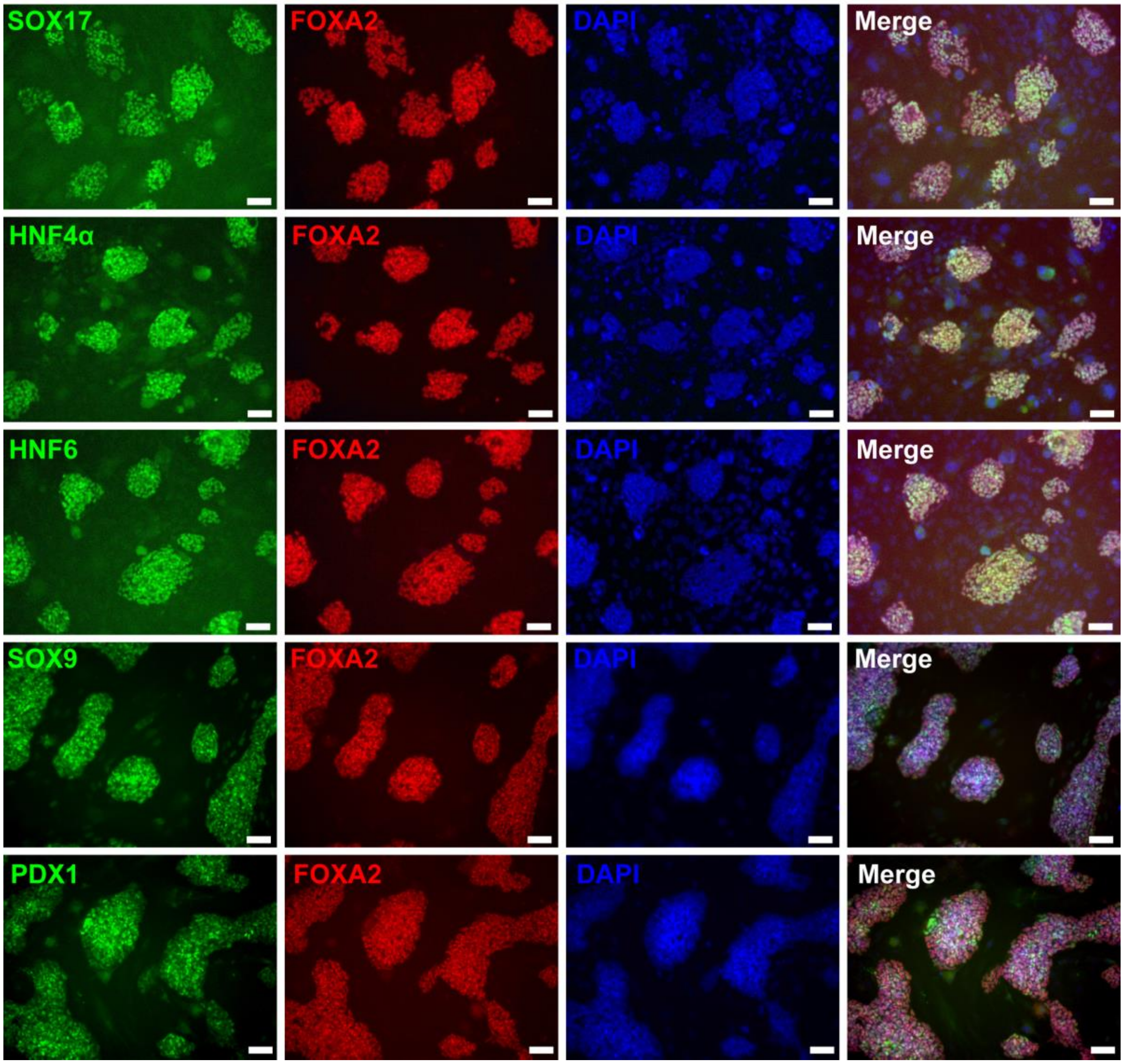

Supplementary Figure 2. Additional characterization of cPF cells. Single channel pictures for the immunostainings shown in Figure 2c. Scale bar, 50 μm.

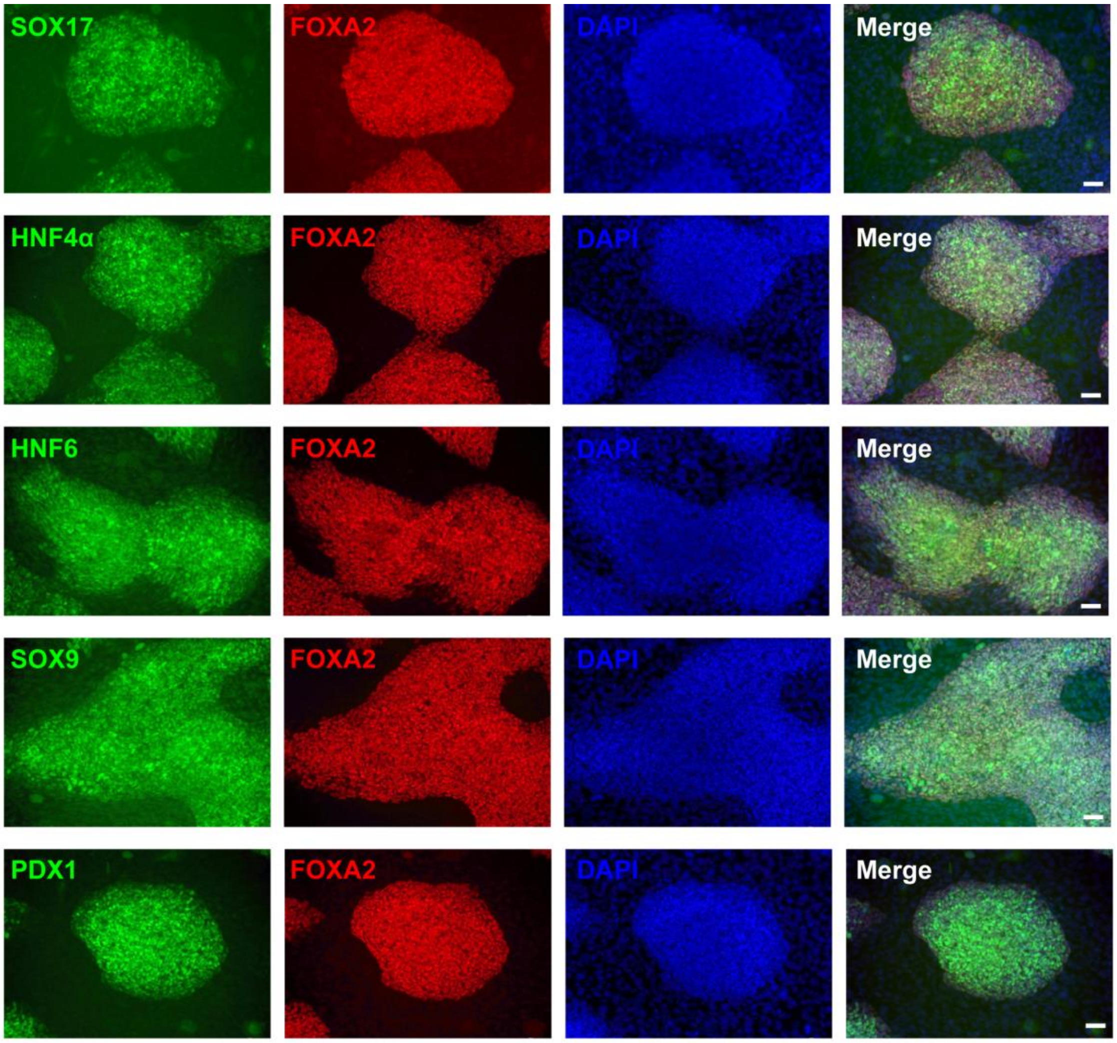

Supplementary Figure 3. Additional characterization of cPF cells. Single channel pictures for the immunostainings shown in Figure 2h. Scale bar, 50 μm.

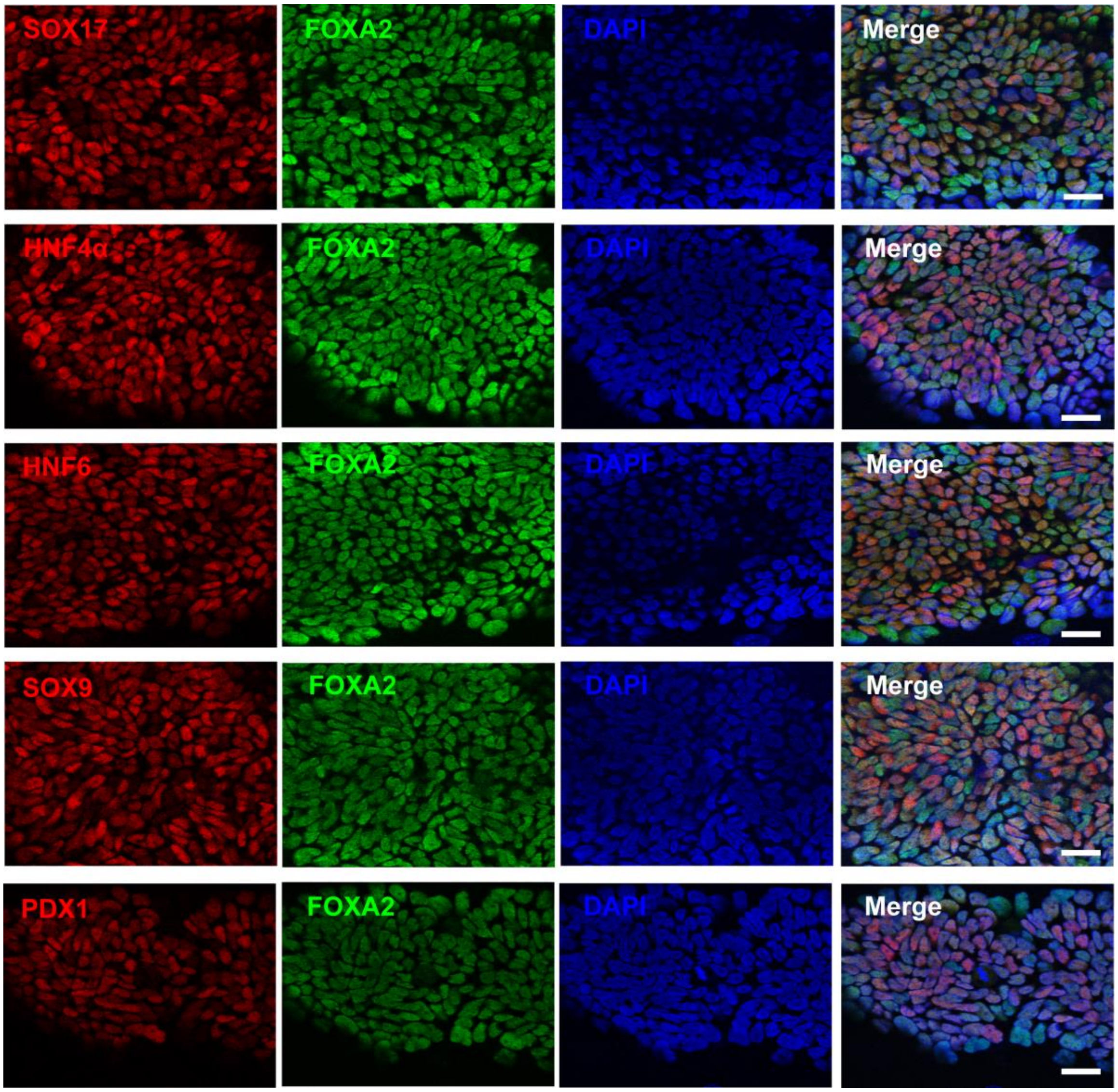

Supplementary Figure 4. Additional characterization of cPF cells. Higher magnification pictures for the immunostainings shown in Figure 2h. Scale bar, 20 μm.

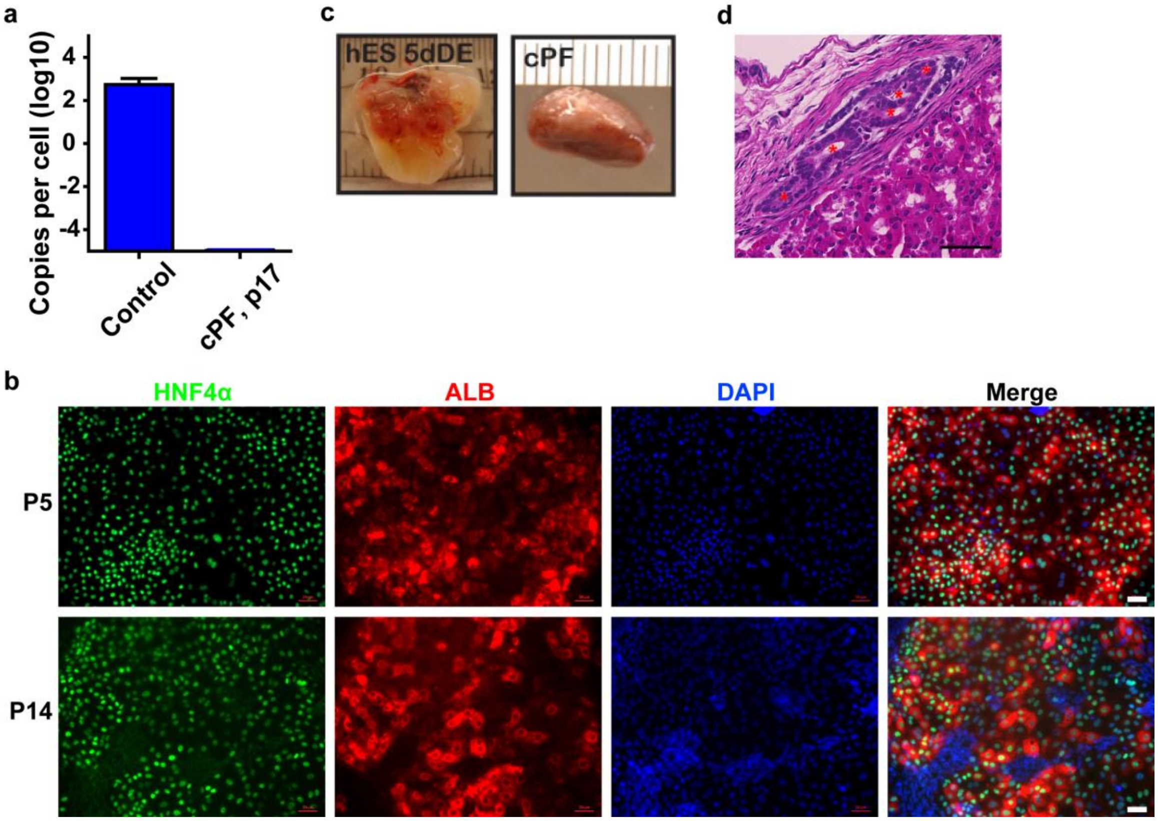

Supplementary Figure 5. Additional characterization of cPF cells. (a) qPCR analysis for the presence of episomal plasmid in established cPF cells at passage 17 (cPF p17). Note that episomal vectors were undetectable by this method in cPF cells. Mean values ± s.e.m. are shown (n=3 experiments). **(b)** Immunofluorescence analysis of p5 and p14 cPF cells differentiated into hepatocytes for the transcription factor HNF4a and Albumin (ALB). Nuclei are visualized by DAPI staining. Scale bar, 50 µm. **(c)** hESC-derived definitive endodermal cells and cPF cells were transplanted under the kidney capsule of immunodeficient mice. None of the cPF cell grafts result in tumor formation, even after prolonged periods of up to 24 weeks *in vivo* (n=10 mice), while hESC-derived endodermal cell grafts (n=4 mice) resulted in tumorigenic structures with cysts and increased graft size already after 7 weeks *in vivo*. **(d)** Representative Haematoxylin and Eosin staining of cPF graft section. Red stars indicated multiple epithelial structures. Scale bar, 50 µm.

a

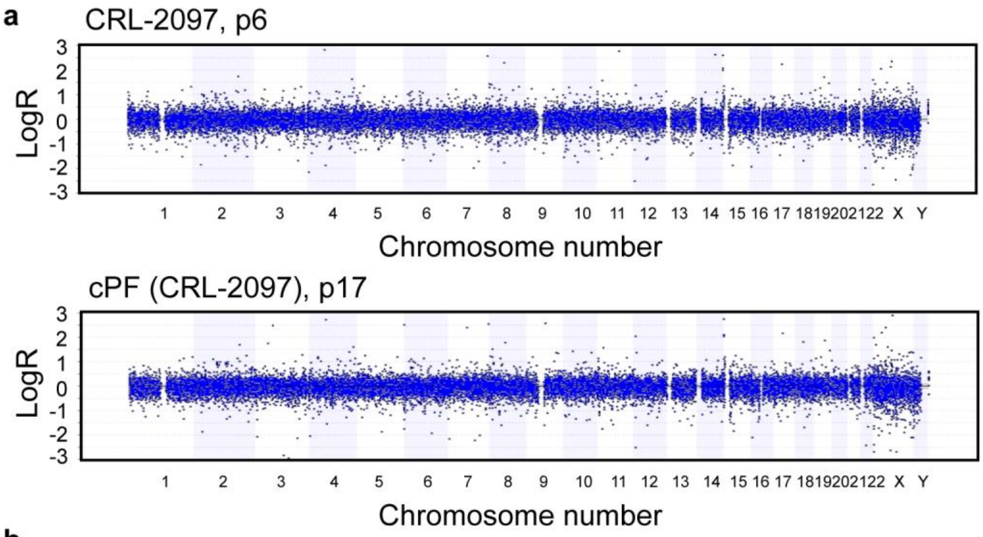

b

Sample	CNV	Location	Type	Annotations
CRL-2097, p6	8	Chr2: 89185302-89301214	Amp	N/A
		Chr4: 69392576-69483277	Del	UGT2B17, UGT2B15
		Chr6: 259316-362290	Del	DUSP22
		Chr8: 39258894-39381514	Del	ADAM5P, ADAM3A
		Chr12: 9637323-9693948	Del	N/A
		Chr14: 106405703-106803307	Amp	ADAM6, NCRNA00226
		Chr14: 106803307-107214893	Amp	NCRNA00221
		Chr15: 22318597-22558756	Del	LOC727924, OR4M2, OR4N4
cPF (CRL-2097), p17	12	Chr2: 89185302-89301214	Amp	N/A
		Chr4: 69392576-69483277	Del	UGT2B17, UGT2B15
		Chr6: 259316-362290	Del	DUSP22
		Chr6: 78979161-79023328	Del	N/A
		Chr8: 39258894-39381514	Del	ADAM5P, ADAM3A
		Chr11: 55385617-55450788	Del	OR4P4, OR4S2, OR4C6
		Chr12: 9637323-9693948	Del	N/A
		Chr14: 19794577-20421677	Amp	POTEM, OR11H2, OR4Q3
		Chr14: 106405703-106803307	Amp	ADAM6, NCRNA00226
		Chr14: 106803307-107214893	Amp	NCRNA00221
		Chr15: 22318597-22558756	Del	LOC727924, OR4M2, OR4N4
		Chr15: 34735949-34785082	Del	N/A

Supplementary Figure 6. Additional characterization of cPF cells. (a+b) Comparative genomic hybridization (CGH) array analysis of parental neonatal fibroblasts (CRL-2097) and established cPF cells at p17. **(a)** No gross chromosomal aberrations were detected in p17 cPF cells compared to parental cells. **(b)** Copy number variation analysis revealed a total of 8 and 12 CNVs in neonatal fibroblasts and expanded cPF cells, respectively. The 4 new CNVs in expanded cPF cells are highlighted in blue.

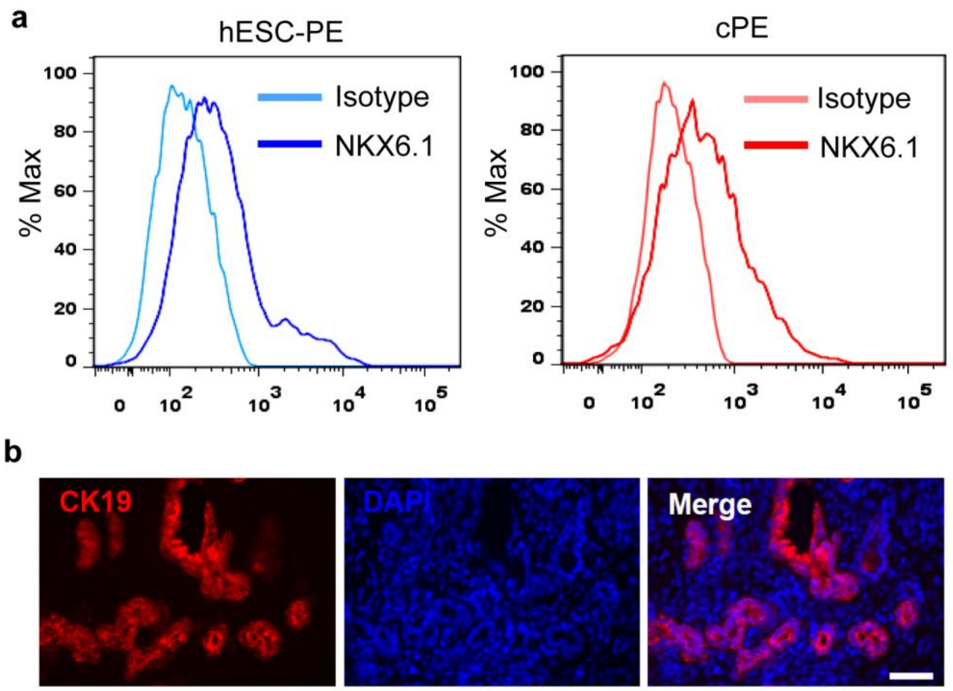

Supplementary Figure 7. Additional characterization of cPE cells. (a) Flow cytometric analysis of NKX6.1 protein expression in cPE cells and hESC differentiated into pancreatic cells. **(b)** Immunofluorescence staining for the duct marker Cytokeratin 19 (CK19) in cPE grafts. Nuclei are visualized by DAPI staining. Scale bar, 50 μm.

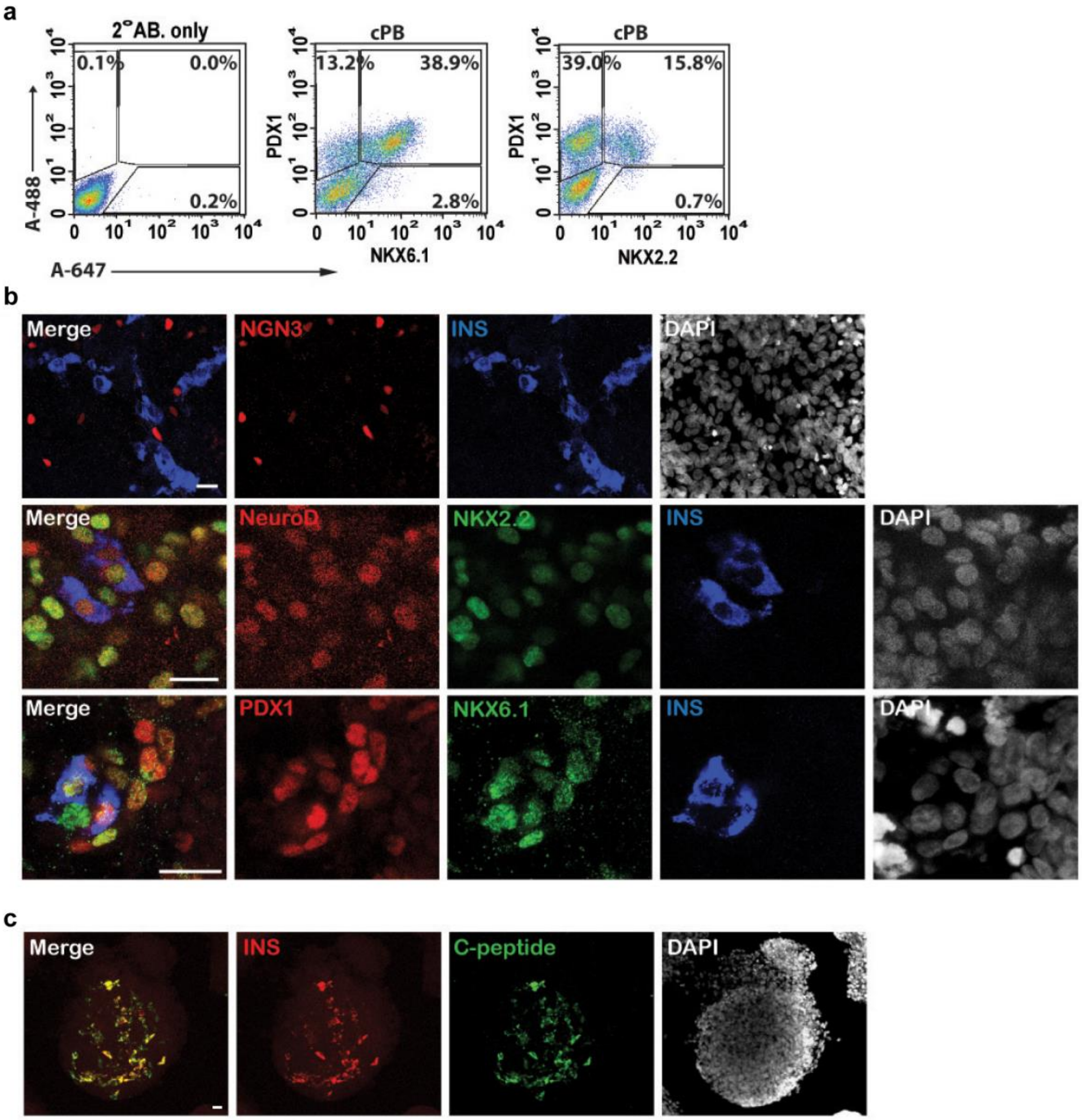

Supplementary Figure 8. Additional characterization of cPB cells. (a) Flow cytometric analysis of PDX1, NKX6.1, and NKX2.2 expression in cPB cells. **(b)** High magnification micrographs of cPB cells. Many of the Insulin (INS) positive cells co-express key beta cell transcription factors PDX1, NKX6.1, NKX2.2, and NEUROD1, but only rarely co-express endocrine progenitor marker NGN3. Scale bar, 20 μm. **(c)** Co-localization of Insulin (INS) and C-peptide staining, excluding Insulin uptake from the media. Scale bar, 20 μm.

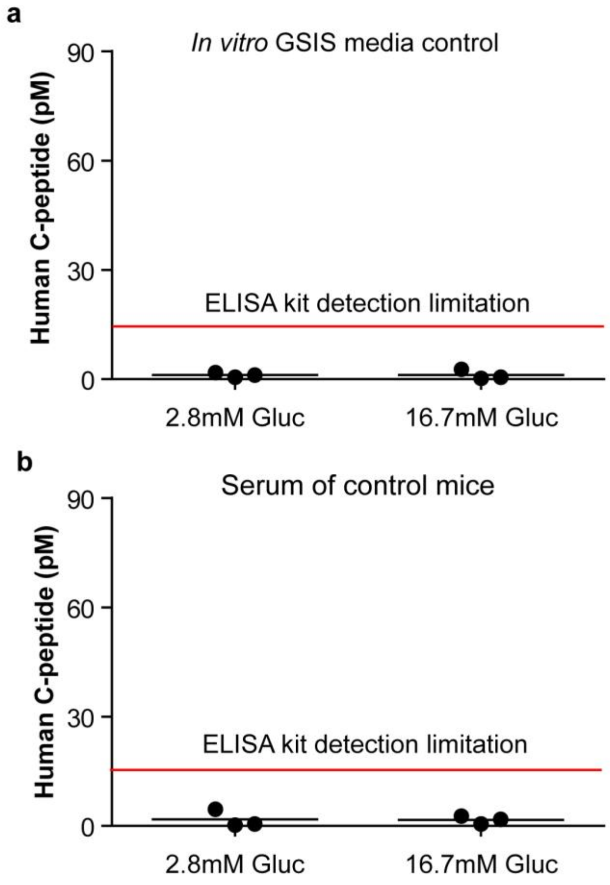

Supplementary Figure 9. Additional characterization of cPB cells. (a+b) ELISA analysis of human C-peptide levels in media employed for *in vitro* GSIS experiment **(a)** and serum from control NSG mice after fast and glucose challenge **(b)**. Note that all data points analyzed were below the detection limit of the assay, indicated by a red line.

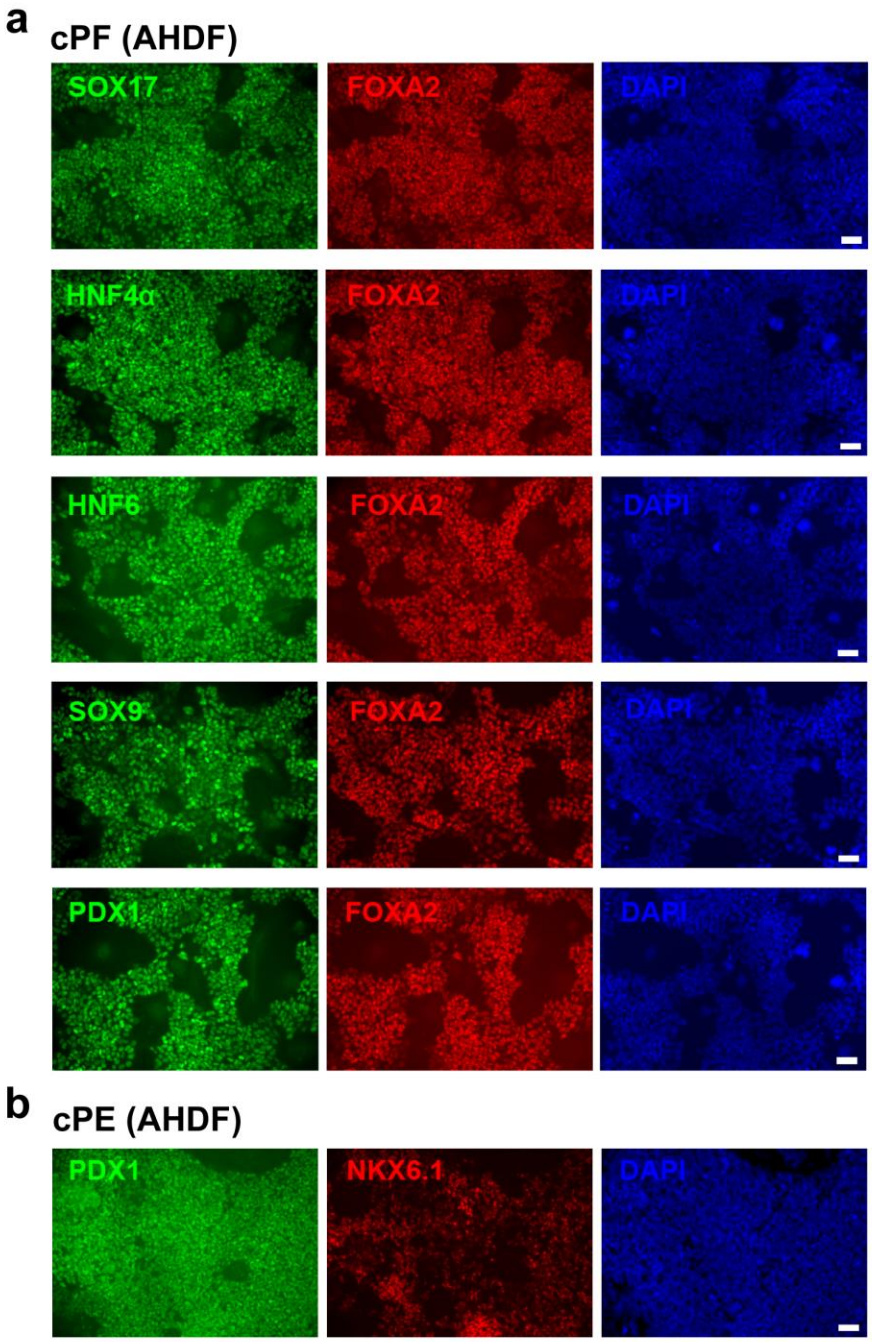

Supplementary Figure 10. Generation of pancreatic beta-like cells from human adult fibroblasts. **(a)** Characterization of cPF cells from human adult fibroblasts by immunofluorescence analysis for SOX17, FOXA2, HNF4A, HNF6, SOX9 and PDX1. Nuclei are visualized by DAPI staining. Scale bar, 50 μm. **(b)** Characterization of cPE cells from human adult fibroblasts by immunostaining of PDX1 and NKX6.1. Nuclei are visualized by DAPI staining. Scale bar, 50 μm.

a

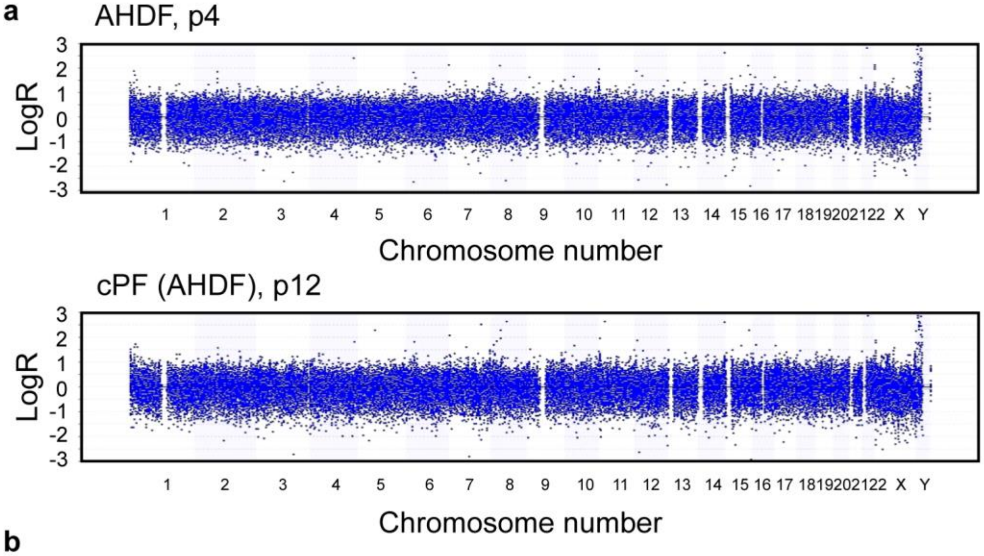

b

Sample	CNV	Location	Type	Annotations
AHDF, p4	6	Chr2: 89141608-89301214	Amp	N/A
		Chr8: 39258894-39381514	Amp	ADAM5P, ADAM3A
		Chr12: 9637323-9693948	Amp	N/A
		Chr14: 106405703-106513022	Amp	ADAM6
		Chr14: 106636701-106957950	Amp	NCRNA00226, NCRNA00221
		Chr22: 23056562-23228483	Amp	MIR650
cPF (AHDF), p12	7	Chr2: 89141608-89301214	Amp	N/A
		Chr8: 39258894-39381514	Amp	ADAM5P, ADAM3A
		Chr12: 9637323-9693948	Amp	N/A
		Chr14: 41616413-41657239	Del	N/A
		Chr14: 106405703-106513022	Amp	ADAM6
		Chr14: 106636701-106957950	Amp	NCRNA00226, NCRNA00221
		Chr22: 23056562-23228483	Amp	MIR650

Supplementary Figure 11. Additional characterization of converted posterior foregut cells (cPF) converted from human adult fibroblasts. (a+b) Comparative genomic hybridization (CGH) array analysis of parental adult fibroblasts (AHDF) and established cPF cells (AHDF) at p12. **(a)** No gross chromosomal aberrations were detected in p12 cPF cells (AHDF) compared to parental cells. **(b)** Copy number variation analysis revealed a total of 6 and 7 CNVs in adult fibroblasts and expanded cPF cells (AHDF), respectively. One new CNV in expanded cPF cells is highlighted in blue.

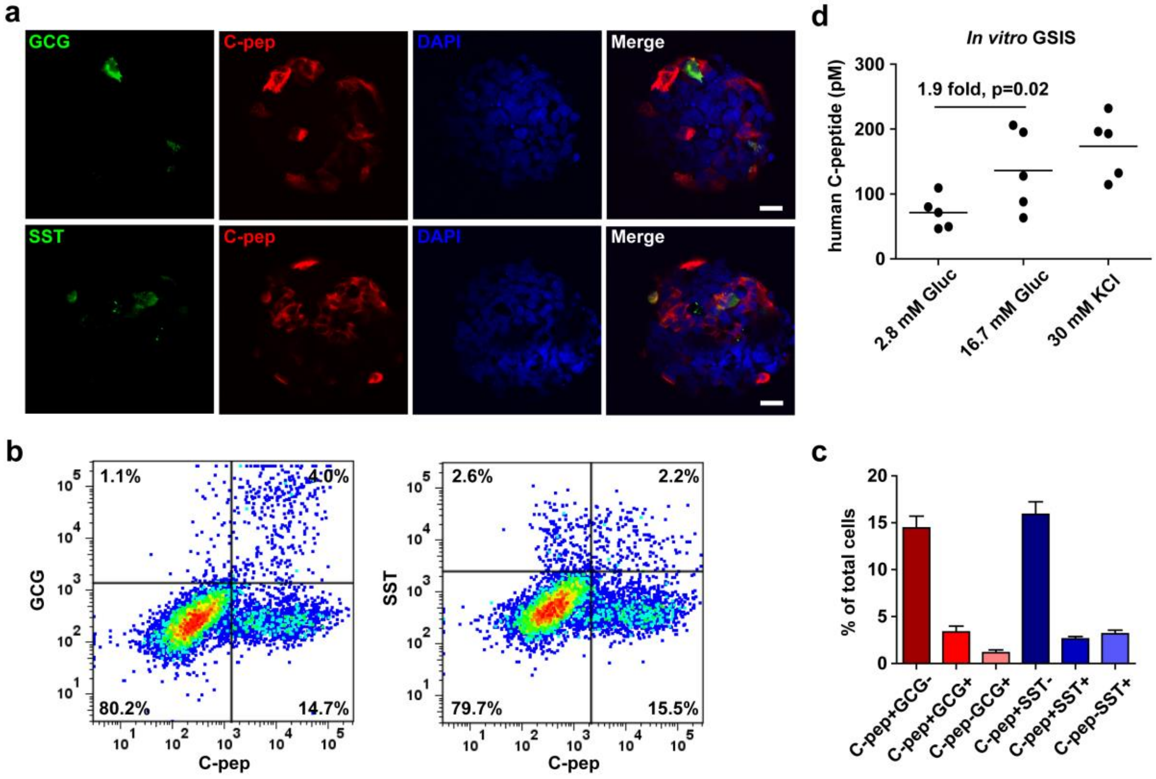

Supplementary Figure 12. Additional characterization of functional pancreatic beta-like cells (cPB) from human adult fibroblasts. (a) Immunofluorescence analysis of cPB cells (ADHF) for C-peptide (C-pep), Glucagon (GCG) and Somatostatin (SST). Scale bar, 20 µm. **(b+c)** Flow cytometric analysis of cPB cells (AHDF) for human C-peptide (C-pep), Glucagon (GCG) and Somatostatin (SST). (**b**) Representative blots for co-staining of human C-pep and GCG or SST are shown. (**c**) Quantification of flow based analysis of the percentage of single and double positive cells for C-pep and GCG or SST. n=3 experiments. **(d)** *In vitro* glucose stimulated insulin secretion (GSIS) assays (n=7 cell cultures of 4 experiments) demonstrated that cPB cells (ADHF) release insulin in response to physiological levels of glucose. Depolarization by higher KCl concentration further increased insulin secretion. Note that insulin release was measured by human specific C-peptide ELISA assay. P-value was calculated using a two-tailed student’s t-test.

Supplementary Table 1. Antibodies used

**Antigen**	**Antibody species**	**Source**	**Dilution**
SOX17	Goat	R&D Systems	1:1000
FOXA2	Goat	R&D Systems	1:1000
FOXA2	Rabbit	Millipore	1:500
HNF4α	Mouse	Perseus Proteomics	1:500
HNF6	Rabbit	Santa Cruz	1:500
PDX1	Goat	R&D Systems	1:1000
NKX6.1	Mouse	Developmental Studies Hybridoma Bank	1:100
SOX9	Rabbit	Santa Cruz	1:500
C-peptide	Rabbit	Abcam	1:200
NANOG	Rabbit	Abcam	1:500
TRA-1-60	Mouse	BioLegend	1:100
NKX2.2	Mouse	Developmental Studies Hybridoma Bank	1:20
NGN3	Sheep	R&D Systems	1:200
NEUROD	Goat	Santa Cruz	1:200
Insulin (INS)	Guinea pig	Dako	1:500
Glucagon (GCG)	Rabbit	Santa Cruz	1:500
Glucagon(GCG)	Mouse	Sigma	1:1000
E-cadherin	Mouse	BD Bioscience	1:200
Beta-catenin	Mouse	BD Bioscience	1:200
HuNu	Mouse	Millipore	1:500
Pan-keratin	Rabbit	Abcam	1:50
HNF4α	Rabbit	Santa Cruz	1:300

C-peptide	Mouse	Millipore	1:200
Somatostatin (SST)	Rabbit	Dako	1:300
CK19	Mouse	Thermo Scientific Lab Vision	1:500
Albumin (ALB)	Goat	Bethyl Laboratories	1:500

Supplementary Table 2. Primer sequences

Genes	Forward primer	Reverse primer
SOX17	GGCGCAGCAGAATCCAGA	CCACGACTTGCCCAGCAT
FOXA2	GGGAGCGGTGAAGATGGA	TCATGTTGCTCACGGAGGAGTA
HNF1A	AACACCTCAACAAGGGCACTC	CCCCACTTGAAACGGTTCCT
HNF1B	CCAAGCCGGTCTTCCATACTC	TGGGAGGTGTGTCATAGTCGT
HNF4A	CTACATCAACGACCGCCAGT	ATCTGCTCGATCATCTGCCAG
HNF6	ATGTCCAGCGTCGAACTCTAC	TGCTTTGGTACAAGTGCTTGAT
PDX1	CCTTTCCCATGGATGAAGTC	CGAACTCCTTCTCCAGCTCTA
NKX6.1	TCAACAGCTGCGTGATTTTC	CCAAGAAGAAGCAGGACTCG
NKX2.2	ATGTAAACGTTCTGACAACT	TTCCATATTTGAGAAATGTTTGC
PTF1A	CATAGAGAACGAACCACCCTTTGAG	GCACGGAGTTTCCTGGACAGAGTTC
HLXB9	TCCACCGCGGGCATGATCCT	GCGCTTGGGCCGCGACAGCTA
NGN3	CCCTCTACTCCCCAGTCTCC	CCTTACCCTTAGCACCCACA
INS	GCAGCCTTTGTGAACCAACAC	CCCCGCACACTAGGTAGAGA
NEUROD	ATGACCAAATCGTACAGCGAG	GTTCATGGCTTCGAGGTCGT
PAX6	TGGGCAGGTATTACGAGACTG	ACTCCCGCTTATACTGGGCTA
RFX6	ATCAGCAGCATTCGTTCACTGC	GGAAGAAGGAATTGGGGTTTGC
MAFA	AGCGAGAAGTGCCAACTCC	TTGTACAGGTCCCGCTCTTT
GCK	GCAGATGCTGGACGACAG	TCCTGCAGCTGGAACTCTG
PCSK1	TGATCCCACAAACGAGAACA	TCTGATTATTTGCTTGCATGG
KIR6.2	TGTGTCACCAGCATCCACTC	CACTTGGACCTCAATGGAGAA
SUR1	AGACCCTCATGAACCGACAG	GGCTCTGTGGCTTTTCTCTC
UCN3	AGGCCTCCCCCACAAGTTCTAC	TTCTCTTTGCCCTCCTCCTCCT

SLC30A8	GAGCGCCTGCTGTATCCTGATT	ATGCACAAAAGCAGCTCTGACG
THY1	ATCGCTCTCCTGCTAACAGTC	CTCGTACTGGATGGGTGAACT
COL1A1	TCACCCACCGACCAAGAAAC	ACGGAAATTCCTCCGGTTGAT
endoOCT4	AGTTTGTGCCAGGGTTTTTG	ACTTCACCTTCCCTCCAACC
endoNANOG	TTTGGAAGCTGCTGGGGAAG	GATGGGAGGAGGGGAGAGGA
SOX1	CAGTACAGCCCCATCTCCAAC	GCGGGCAAGTACATGCTGA
BYACHYUARY	AATTGGTCCAGCCTTGGAAT	CGTTGCTCACAGACCACA
GAPDH	TGCACCACCAACTGCTTAGC	GGCATGGACTGTGGTCATGAG
